# A noncanonical response to replication stress protects genome stability through ROS production, in an adaptive manner

**DOI:** 10.1038/s41418-023-01141-0

**Published:** 2023-03-03

**Authors:** Sandrine Ragu, Nathalie Droin, Gabriel Matos-Rodrigues, Aurélia Barascu, Sylvain Caillat, Gabriella Zarkovic, Capucine Siberchicot, Elodie Dardillac, Camille Gelot, Josée Guirouilh-Barbat, J. Pablo Radicella, Alexander A. Ishchenko, Jean-Luc Ravanat, Eric Solary, Bernard S. Lopez

**Affiliations:** 1 Université de Paris, INSERM U1016, UMR 8104 CNRS, Institut Cochin, 24 rue du Faubourg St. Jacques, 75014 Paris, France; 2 CNRS UMR 8200, Institut de Cancérologie Gustave-Roussy, Université Paris Saclay, 114 Rue Edouard Vaillant, 94805 Villejuif, France; 3 Institut de Cancérologie Gustave-Roussy, INSERM U1287, Université Paris Saclay, Equipe Labellisée Ligue Contre le Cancer 114 Rue Edouard Vaillant, 94805 Villejuif, France; 4 IRIG/DIESE/SyMMES/CIBEST, UMR 5819 CEA/CNRS/UGA, CEA Grenoble, 38054 Grenoble, France; 5 Group “Mechanisms of DNA Repair and Carcinogenesis”, Gustave Roussy Cancer Campus, CNRS UMR9019, Université Paris-Saclay, 94805 Villejuif, France; 6 Université Paris-Saclay, Université Paris-Cité, CEA/IRCM. UMR Stabilité Génétique Cellules Souches et Radiations, F-92260 Fontenay-aux-Roses, France; 7 Present Address: Institut de Biologie Physico-Chimique, Paris, 75005 France; 8 Present Address: Institut Curie, Paris, 75005 France

**Keywords:** Gene regulation, Molecular biology, Cell biology, Genetics

## Abstract

Cells are inevitably challenged by low-level/endogenous stresses that do not arrest DNA replication. Here, in human primary cells, we discovered and characterized a noncanonical cellular response that is specific to nonblocking replication stress. Although this response generates reactive oxygen species (ROS), it induces a program that prevents the accumulation of premutagenic 8-oxoguanine in an adaptive way. Indeed, replication stress-induced ROS (RIR) activate FOXO1-controlled detoxification genes such as *SEPP1, catalase, GPX1*, and *SOD2*. Primary cells tightly control the production of RIR: They are excluded from the nucleus and are produced by the cellular NADPH oxidases *DUOX1/DUOX2*, whose expression is controlled by NF-κB, which is activated by PARP1 upon replication stress. In parallel, inflammatory cytokine gene expression is induced through the NF-κB-PARP1 axis upon nonblocking replication stress. Increasing replication stress intensity accumulates DNA double-strand breaks and triggers the suppression of RIR by p53 and ATM. These data underline the fine-tuning of the cellular response to stress that protects genome stability maintenance, showing that primary cells adapt their responses to replication stress severity.

## Introduction

Cells are continually challenged by exogenous as well as endogenous assaults that can compromise genome stability, ultimately leading to cell death, inflammation, premature aging and oncogenesis. Indeed, genome instability is a hallmark of cancer and aging cells [1–3]. To counter these stresses, the DNA damage response (DDR) coordinates a network of pathways ensuring faithful genome transmission. Defects in the DDR result in sensitivity to genotoxic agents, genome instability, and neuronal defects and are frequently associated with cancer predisposition and premature aging [1, 4–9]. In particular, the DDR is activated at the pre/early steps of senescence and tumorigenesis [4–6, 10, 11].

Following genotoxic stress, the activation of the DDR leads to the arrest of cell cycle progression at the G_1_-S, intra-S and G_2_-M “cell cycle checkpoints” before engaging the replication and mitosis phases, which are sensitive to genome stability [8]. It is generally thought that this cell cycle arrest allows both sufficient time and full accessibility to essential cofactors (ATP and nucleotides) to the DNA repair/recombination machinery to repair damaged DNA. *In fine*, this coordinated response allows the surviving cells to resume replication with an intact DNA matrix [8, 12, 13]. However, even in the absence of exogenous stress, cells are still routinely subjected to inevitable endogenous stresses, such as replicative stress and oxidative stress, which jeopardize genome integrity. Indeed, replication fork progression is spontaneously hampered by endogenous hindrances (structures that are difficult to replicate, conflicts with transcription, proteins that are tightly bound to DNA, endogenous damage, etc.) [14–17]. In addition, reactive oxygen species (ROS), which are spontaneously generated as byproducts of cell metabolism, can alter replication dynamics [18–20]. Despite continuous exposure to chronic endogenous stresses, cells continue to proliferate and replicate their genome, suggesting that the DDR is not or is not completely activated and thus that such nonblocking endogenous stresses should be of “low” intensity. This raises the question of whether cells actually respond to low-level stresses or have developed specific alternative response(s).

While ROS generate DNA damage, stressed cells exposed to DNA damaging agents reciprocally produce ROS [21–23]. In particular, hydroxyurea (HU), an inhibitor of ribonucleotide reductase that generates replication stress, has been shown to induce ROS [24, 25]. However, the impact of HU on ROS production remains poorly documented in mammalian cells. Stress-induced ROS are generally interpreted as byproducts of the cell response to stress; for instance, respiratory chains produce ATP and ROS as byproducts, especially under severe stresses that demand substantial energy (thus ATP) to be managed. Notably, unchallenged cells from patients with DDR syndromes frequently exhibit spontaneously increased levels of endogenous ROS. In addition, cells with deficient homologous recombination (HR) leading to altered replication dynamic processes [26, 27] also exhibit spontaneously high levels of ROS [18]. This finding suggests that even low-level/endogenous stresses, i.e., below those that trigger full DDR activation and cell cycle arrest, can induce ROS production in mammalian cells. Considering that ROS can also serve as secondary messengers in different biological cell pathways [28–31], here, we addressed the question of whether ROS production might represent a component of an autonomous cellular response to low-level/endogenous genotoxic stress. Given that replication stress is a primary endogenous stress, we analyzed the impact of different replication stress intensities and different replication stress inducers on the cell-controlled production of ROS.

Here, we show that primary human cells respond to replicative stress in two distinct phases, adapting the response to stress severity. In primary human fibroblasts, low replicative stress that does not lead to full replication arrest induces ROS production controlled by the cellular NADPH oxidases DUOX1 and DUOX2, controlled by NF-κB, which is activated by the PARP1 protein. This cell-controlled replication stress-induced ROS (RIR) production prevents the accumulation of premutagenic DNA lesions 8-oxoguanine (8-oxoG) through the induction of the FOXO1 detoxification pathway that induces detoxifying genes, such as *SEPP1, catalase, GPX1*, and *SOD2*. This response also protects cells from exogenous exposure to hydrogen peroxide, defining an adaptive-like response to low doses. Notably, the treatment of chronic myelomonocytic leukemia (CMML) patients with hydroxyurea (HU) activates the NF-κB and FOXO1 pathways in proliferating cells, revealing the activation of this pathway in vivo. Increasing replication stress generates the accumulation of DNA double strand breaks, arrests DNA synthesis and suppresses RIR. These data highlight that the cellular response to replication stress can be subdivided into two phases: a low-level stress DDR (LoL-DDR), which is adaptive, protecting against the accumulation of premutagenic lesions, and a high-level stress DDR, which arrests replication. Therefore, RIR appears to be the outcome of an autonomous response that is tightly controlled by the cell, not merely a passive response. These data reveal a specific cellular defense response to low-level/endogenous stress, underlining the fine-tuning of the cellular responses to stress severity.

## Results

### Human primary fibroblasts produce ROS as a cell autonomous response specific to nonblocking replication stress

**Fig. 1:**
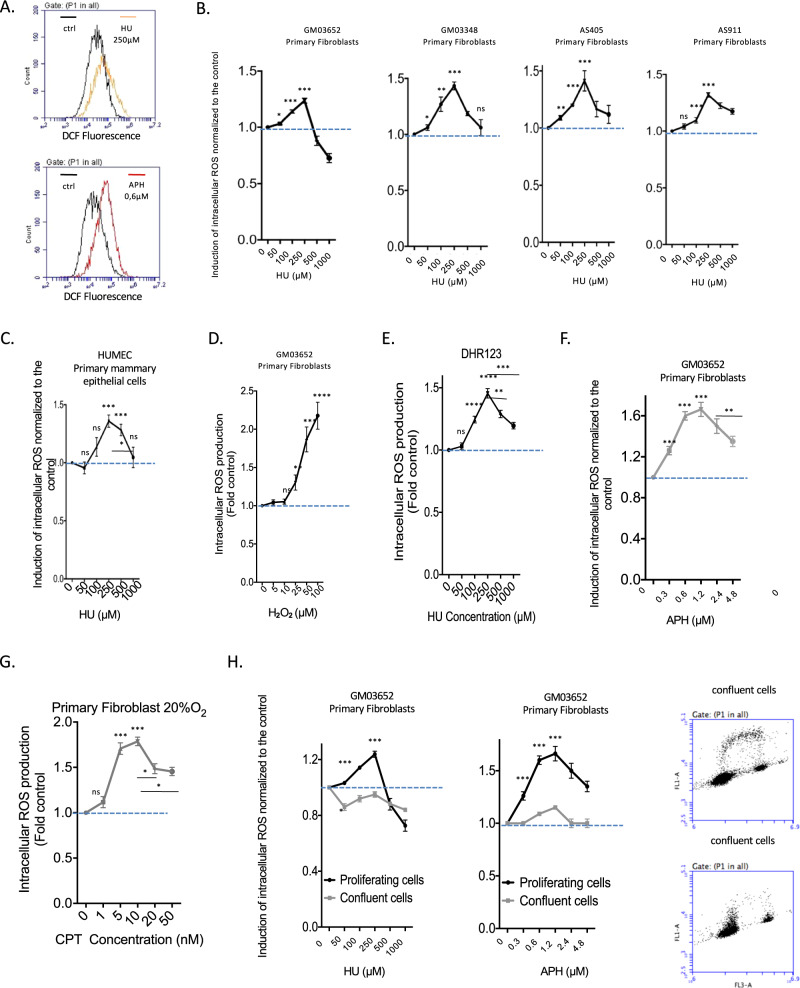
Primary cells induce ROS upon nonblocking replication stress. **A** HU- or APH-induced ROS production in primary human fibroblasts was monitored using the DCFDA fluorescent probe and FACS analysis. The shift in the fluorescence peak reveals the induction of intracellular ROS production. **B** HU dose-dependent induction of ROS production using the DCFDA fluorescent probe in four different primary fibroblast strains. **C** HU dose response of RIR production in primary mammary epithelial cells (HUMEC). **D** H_2_O_2_ dose response of ROS production measured with the DCFDA fluorescent probe and FACS analysis. The data from three independent experiments are presented as the mean (± SEM) level of ROS production normalized to that of the control. **E** HU-induced ROS monitored with a different fluorescent probe: DHR (dihydrorhodamine 123). **F** Impacts of aphidicolin (APH) on ROS production in primary fibroblasts. **G** Impact of camptothecin (CPT) on the production of ROS in primary fibroblasts. Data from three independent experiments are presented as the mean ( ± SEM) level of ROS production normalized to that of the control. **H** Cell confluence abrogates HU- and APH-induced ROS production (left panel). Data from three independent experiments are presented as the mean ( ± SEM) level of ROS production normalized to that of the control. Right panel: Measurement of the impact of confluence on DNA replication monitored by BrdU incorporation and measured by FACS analysis.

To investigate the impact of replication stress on the production of ROS, i.e., RIR, we first used a 2’,7’-dichlorofluorescein diacetate (DCFDA) fluorescent probe that monitors the intracellular ROS level (Fig. 1A). This method also enables easy analyses of the dose-response and kinetics. We treated 4 different strains of primary human skin fibroblasts and one primary mammary epithelial cell strain (HUMEC) with increasing doses of HU (Fig. 1B, C).

All 4 primary strains consistently responded identically (Fig. 1B, C): a slight induction was recorded at low HU doses, reaching a peak at 250 µM HU; then, ROS production decreased at higher doses (Fig. 1B, C; Fig. S1). Although these inductions were moderate, they were statistically significant and reproducible. Indeed, peak-shaped dose response curves were consistently observed in all 4 different human primary fibroblast strains and in each of the individual experimental repeats. In addition, the peak RIR level was always observed at the same dose, i.e., 250 µM HU (Fig. 1B, C, Fig. S1). The level of ROS produced at the peak (exposure to 250 µM HU) was comparable to that produced upon exposure to 25 µM H_2_O_2_ (compare Fig. 1B–D). Moreover, ROS were also detected with the same peak-shape dose response curve at the same doses using another fluorescent probe (dihydrorhodamine 123, DHR) (Fig. 1E). The production of ROS was confirmed by exposure of the cells to the antioxidant N-acetyl-cysteine (NAC), which abolished RIR (Fig. S2A) without affecting cycle progression or DNA synthesis (Fig. S2B). Note that RIR was induced in cells maintained in 3% and 20% oxygen (Fig. S2C), confirming that the induction of cellular RIR production per se did not depend on the level of ambient oxygen.

Taken together, these data attest to the robustness of this response and suggest that it should correspond to an actual cell-autonomous response to low-level replication stress.

Then, we detailed RIR in primary fibroblasts. RIRs were clearly observable after 3 days of exposure to 250 µM HU and could be maintained for several days (Fig. S3). These data are consistent with the fact that a sufficient number of cells should reach S phase (see below) and that the division time of primary fibroblasts is between 30 and 48 h. Therefore, for all the experiments (above and below), we chose to treat the primary cells with HU for 3 days. This time period is sufficient to detect significant levels of RIR with a non-significant increase in senescent cells, while one week of exposure induces a significant increase in senescent cells, as detected by the β-galactosidase assay.

When using a different replication stress inducer, aphidicholin (APH), the dose–response curve shapes were similar to those obtained with HU (Fig. 1F). A third replication stress inducer, the topoisomerase I inhibitor camptothecin (CPT), also generated peak-shaped ROS production in primary human fibroblasts (Fig. 1G). These data support that ROS production actually results from replication stress. Consistent with this conclusion, RIR production was abolished in confluent cells, i.e., nonreplicating cells (Fig. 1H). This result shows that the production of ROS depends on the state of cell proliferation, confirming that it constitutes a response to replication stress.

Of note, a HU dose (250 µM) or APH dose (0.6 µM) that induced the peak of RIR did not significantly affect DNA synthesis or the cell cycle distribution of primary human fibroblasts (Figs. S4A and S4B). Given that BrdU was efficiently incorporated into treated cells (at levels similar to those in nontreated cells), many cells sustained DNA synthesis at these doses (Figs. S4A and S4B). However, low doses of HU have been shown to reduce replication fork velocity [26, 32]. Therefore, at the HU doses that induce RIR, replication is likely slowed but not arrested. In contrast, at higher HU doses (1 mM HU), which do not induce RIR, many cells are blocked in S phase and do not incorporate BrdU (Fig. S4A). At such a high HU dose (1 mM), cells that still incorporated BrdU accumulated in early S phase (Fig. S4A). These latter data are consistent i) with a strong slowing down of replication fork velocity and ii) with the fact that HU blocks elongation rather than the initiation of replication [33, 34]. Collectively, these data link RIR production to DNA replication. However, our data show that RIR in primary fibroblasts are produced at doses that do not block replication, and not at high doses that strongly arrest replication; therefore, the RIR in primary fibroblasts correspond to a response specific to “low”, i.e., nonblocking, replication stress.

To extend and confirm the production of ROS by “low” (nonblocking) replication stress, with another method, we used a plasmid encoding an engineered GFP (Ro1 pEGFP-N1) that becomes fluorescent upon oxidation [35]. This method also allowed us to analyze the subcellular localization of RIR in mammalian cells.

**Fig. 2:**
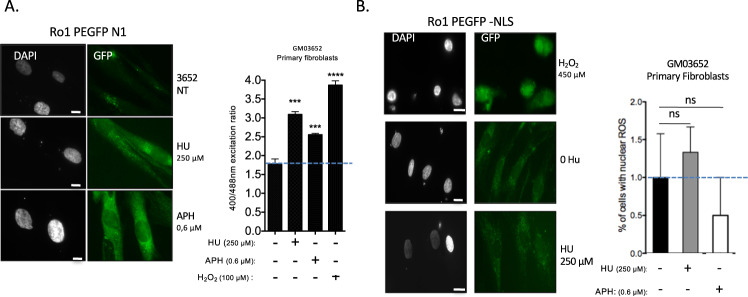
RIR production by different replication stress inducers. **A** Oxidation of ro1pEGFP-N1 expressed in primary fibroblasts exposed to HU or APH. Cells were treated with HU (250 µM; 3 days), APH (0.6 µM; 3 days) or H_2_O_2_ (100 µM; 20 min) as a positive control. Left panel: fluorescence of ro1pEGFP-N1 (480 nm excitation) in primary cells exposed to HU or APH. Right panel: Ratio of fluorescence at 400/480 nm excitation; the histogram represents the mean ± S.E.M. of four independent experiments. *p < 0.01 *vs*. control, determined by the t-test. **B** ROS induced by HU or APH are excluded from the nucleus in primary fibroblasts. Left panels: representative image of GFP fluorescence. The positive control (H_2_O_2_) exhibited GFP fluorescence in the nucleus. Right panel: quantification of cells with nuclear ROS (GFP fluorescence in the nucleus). The histogram represents the mean ± S.E.M. (normalized to the control) of four independent experiments. Ns Not significant *vs*. control as determined by the t-test.

As a positive control, the pro-oxidant hydrogen peroxide (H_2_O_2_) increased the fluorescence ratio when excited at 400/488 nm (Fig. 2A). Cells treated with replication stress inducers, namely, HU or APH at the dose that generated the peak RIR (see Fig. 1B and F), also exhibited an increased *GFP* fluorescence ratio (400/488 nm) (Fig. 2A). As a control, we verified that treatment with these doses of HU did not affect the efficiency of plasmid transfection (Fig. S5).

To monitor the presence of ROS in the nucleus, we used a GFP probe containing a nuclear localization signal. No increased fluorescence was detected in the nuclei of primary fibroblasts, and only a faint signal was detected in the cytoplasm (Fig. 2B). As a positive control, H_2_O_2_, which generated nuclear ROS, showed that the reporter transgene (NLS-GFP) was expressed and able to monitor nuclear ROS in primary fibroblasts (Fig. 2B).

In mammalian cells, alteration of the nucleotide pool has been shown to generate nuclear ROS [25]. We show here that ROS production is in fact linked to replication stress, but RIR localization is restricted to the cytoplasm in primary cells.

### RIR prevents primary fibroblasts from accumulating oxidative DNA lesions

**Fig. 3:**
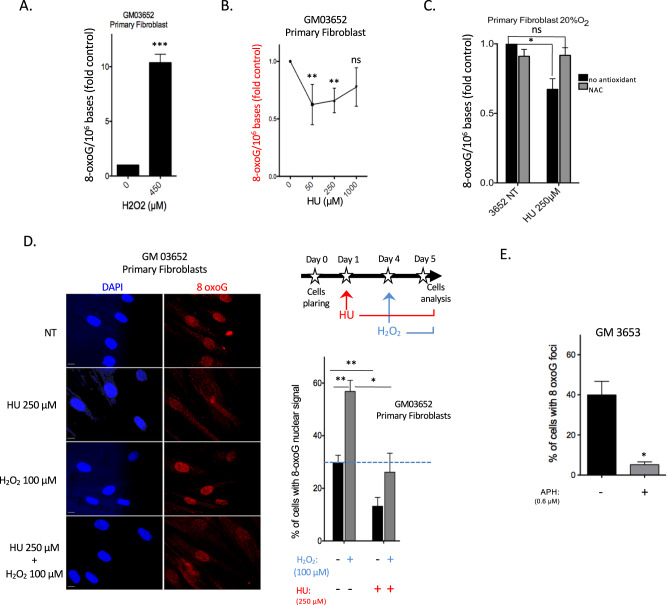
RIR prevents the accumulation of genomic 8-oxoG. **A** Accumulation of 8-oxoG in the genome of primary fibroblasts exposed to 450 µM H_2_O_2_ for 90 min (positive control). **B** 8-OxoG levels in the genome of primary fibroblasts after 72 h of exposure to HU. Quantification of 8-oxoG (8-oxoG/million bases). **C** Effect of NAC on 8-oxoG levels in the genome of primary fibroblasts. **D** 8-OxoG-positive primary fibroblasts upon 72 h exposure to HU using an antibody raised against 8-oxoG. Left panel: representative photos of immunofluorescence staining for 8-oxoG (red) in primary fibroblasts. Scale bars: 10 µm. Upper right panel: scheme of the experimental protocol; 24 h after plating, HU (250 µM) was added to the cells and maintained for the rest of the experiment; 24 h after HU pretreatment, cells were exposed to H_2_O_2_ (100 µM H_2_O_2_); 24 h after H_2_O_2_ treatment, cells were fixed for analysis with the anti-8-OxoG antibody. Lower right panel: Quantification of the frequency of HU-pretreated primary fibroblasts exposed to nuclear localization of 8-oxoG. At least 200 cells were counted. **E** Quantification of the frequency of primary fibroblasts treated with 0.6 µM APH for 72 h, with nuclear localization of 8-oxoG. The histogram represents the mean ± S.E.M. (normalized to the control) of four independent experiments. *p < 0.01 *vs*. control as determined by the *t*-test.

We then assessed whether RIR might impact the accumulation of oxidative damage in the nuclear genome of primary cells. To address this question, we quantified the main premutagenic oxidized base lesion, 8-oxo-guanine (8-oxoG), which commonly serves as a marker of genotoxic oxidative stress, using isotope dilution high-performance liquid chromatography coupled with electrospray ionization tandem mass spectrometry (HPLC–MS/MS) [36]. As a control, H_2_O_2_, which generates nuclear ROS (see Fig. 1D), led to high amounts of 8-oxoG in genomic DNA (Fig. 3A). Although HU treatment increased the intracellular level of ROS (see above), it did not increase the frequency of genomic 8-oxoG in primary fibroblasts (Fig. 3B), consistent with the absence of nuclear RIR (compare Figs. 2B and 3B). Strikingly, the lowest HU doses (50 and 250 µM) significantly decreased the frequency of genomic 8-oxoG, whereas the highest dose (1 mM) neither decreased nor increased the frequency of genomic 8-oxoG (Fig. 3B, C). Remarkably, the doses of HU that generated RIR ( ≤ 250 µM) corresponded to those that protected against the accumulation of 8-oxoG (compare Figs. 1B and 3B), whereas the highest dose (1 mM) that did not induce RIR production did not prevent 8-oxoG accumulation (compare Fig. 1B and Fig. 3B). These findings reveal a paradoxical correlation between the production of RIR and protection against 8-oxoG accumulation.

These results suggest a physiological role for RIR in primary cells, namely, genome protection from the accumulation of premutagenic oxidative DNA alterations.

To determine whether RIR actually protects the genome from 8-oxoG accumulation, we examined the impact of the antioxidant NAC. We predicted that NAC might have opposite effects on 8-oxoG accumulation: 1) a decrease in basal endogenous ROS levels will lead to a decrease in endogenous genomic 8-oxoG frequency, and 2) in contrast, based on the above results, the abrogation of RIR through exposure to NAC (see Fig. S2A) will suppress the protection they provide against 8-oxoG accumulation. Unstressed cells exposed to NAC exhibited a slight decrease in 8-oxoG frequency (Fig. 3C). In HU-exposed cells, NAC treatment abolished the substantial decrease in 8-oxoG accumulation, with 8-oxoG levels becoming similar to those of the unchallenged control cells (Fig. 3C). These data show that the abrogation of RIR suppresses their protection against the accumulation of 8-oxoG.

To confirm these data, we used an anti-8-oxoG antibody. The frequency of 8-oxoG-positive cells decreased upon HU (Fig. 3D) or APH treatment (Fig. 3E). As a positive control, exposure to H_2_O_2_ significantly increased the frequency of 8-oxoG-positive cells (Fig. 3D). Remarkably, pretreatment with HU prevented this stimulation (Fig. 3D).

Thus, in primary fibroblasts, RIR prevents the accumulation of endogenous premutagenic 8-oxoG lesions in the genome and, in addition, prevents the accumulation of 8-oxoG generated by an exogenous pro-oxidant. Therefore, RIR induces an actual program that protects cells against different sources of ROS.

Taken together, these data support the concept that RIR corresponds to an adaptive autonomous cellular response to low-level replication stress.

### RIR activates the FOXO1 detoxification pathway

Although low-level/endogenous stress promotes ROS (RIR) production, this leads to a reduced level of 8-oxoG in the genome. Two hypotheses could explain this apparent paradox: either RIR induces DNA repair mechanisms, or an ROS detoxification process is activated, leading to an adaptive response. Immunoblot analysis of HU-treated cell extracts (Fig. S6A) showed that at the lowest HU doses (50 and 100 µM HU), the level of OGG1, which repairs 8-oxoG, was not increased but was, in contrast, slightly decreased. Despite this downregulation of OGG1 expression, the levels of 8-oxoG decreased at these doses (compare Fig. S6A and Fig. 3B, C). At the highest HU doses, although the OGG1 levels increased, the level of 8-oxoG remained unchanged (compare Fig. S6B and Fig. 3B). Therefore, OGG1 levels cannot explain the variations in genomic 8-oxoG accumulation following HU. The level of MTH1, which removes oxidized nucleotides from nucleotide pools, did not increase but slightly decreased at all HU doses (Fig. S6A) and thus cannot account for the decrease in genomic 8-oxoG at the lowest HU doses (see Fig. 3B, C). In vitro repair assays revealed that neither pAPE1 nor OGG1 activity was induced by HU treatment, with OGG1 activity instead being decreased (Fig. S6B and S6C). Collectively, these data do not support the induction of the DNA repair machinery by HU.

**Fig. 4:**
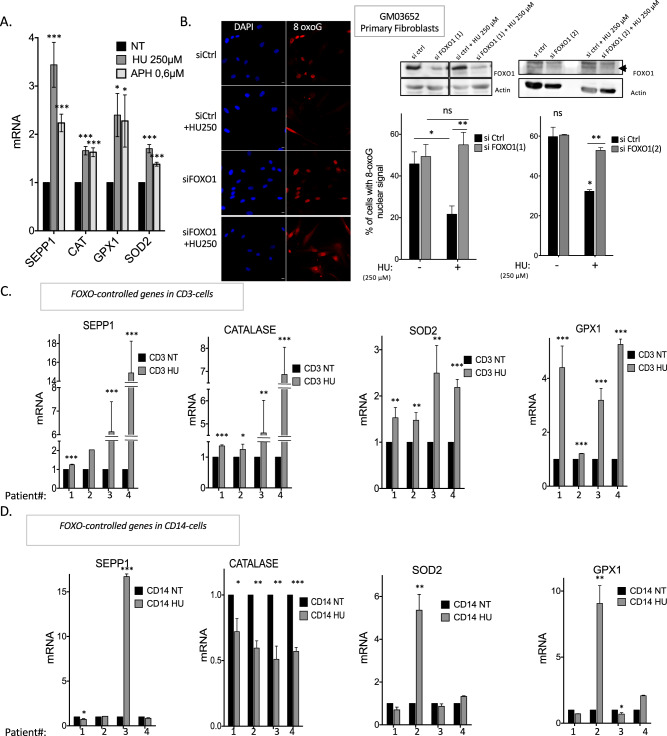
RIR protects primary fibroblasts from endogenous premutagenic oxidative DNA lesions through FOXO1 activation. **A** RIR-inducing doses of HU increase the mRNA levels of 4 different detoxification genes (*SEPP1, catalase, GPX1*, and *SOD2*) controlled by FOXO1 in primary fibroblasts. **B** Impact of silencing *FOXO1* on the frequency of 8-oxoG-positive cells (primary fibroblasts) upon 72 h of exposure to HU. Two siRNAs were used: FOXO1(1) and FOXO1(2). Left panel: immunofluorescence staining for 8-oxoG (red) in primary fibroblasts: representative photos of immunofluorescence staining in primary fibroblasts. Nuclei were counterstained with DAPI (blue). Scale bars: 10 µm. Upper right panel: immunoblot of *FOXO1* silencing in primary fibroblasts. Lower right panels: quantification of the frequency of nuclear 8-oxoG-positive cells upon exposure to 250 µM HU after *FOXO1* silencing. The data from four independent experiments are presented. At least 200 cells were counted*.* **C** Four FOXO-controlled genes (namely, *SEPP1, catalase, SOD2* and *GPX1*) were induced in CD3-positive T cells by HU therapy in CMML patients. **D** Four FOXO-controlled genes (namely, *SEPP1, catalase, SOD2*, and *GPX1*) were not systematically induced in nonproliferating CD14-positive cells.

Using a candidate approach, we monitored the expression of ROS detoxification genes controlled by the ROS-inducible transcription factors FOXO1 and NRF2. The expression of 4 detoxification genes (*SEPP1, catalase, GPX1*, and *SOD2*) that are all controlled by FOXO1 was induced by RIR-inducing doses of HU (Fig. 4A). Notably, in most cases, the corresponding protein level was also induced (Fig. S7). Although the expression of NRF2-controlled genes remained unchanged upon HU treatment in cultured primary human fibroblasts (Fig. S8), we cannot definitively exclude that NRF2 could be activated in other cell types or tissues. To test the involvement of the FOXO1 pathway in the reduction of the 8-oxoG-positive cell frequency by RIR, we silenced *FOXO1* expression by siRNA. Our data show that abrogation of *FOXO1* expression in HU-treated cells restored the frequency of 8-oxoG-positive cells to that of control cells not exposed to HU (Fig. 4B). In conclusion, *FOXO1* contributes to the LoL-DDR, protecting cells from the accumulation of genomic 8-oxoG: RIR activates the expression of FOXO1-controlled genes, leading to lower levels of genomic 8-oxoG accumulation in an adaptive manner.

### FOXO1-controlled genes are induced in chronic myelomonocytic leukemia patients treated with HU

To test the activation of *FOXO1*-controlled genes in vivo, we took advantage of patients suffering from chronic myelomonocytic leukemia (CMML) and treated with HU. CMML, a severe clonal hematopoietic malignancy, is the most frequent myelodysplastic syndrome/myeloproliferative neoplasm. Most patients receive symptom-adapted treatments, such as HU, during the most proliferative stages of the disease [37]. Remarkably, the HU concentrations used herein cultured primary fibroblasts are in the range of those measured in the serum of patients who receive 1000 mg HU per day orally [38, 39]. HU treatment aims to reduce the number of circulating myeloid cells, which may be obtained by decreasing progenitor and precursor proliferation without inhibiting this proliferation, which would be deleterious for the patient. Therefore, we tested whether such treatment activates the expression of *FOXO1*-controlled genes in vivo in a physio-pathological context.

Peripheral blood samples were collected before and after treatment initiation, and gene expression was analyzed in proliferative CD3-positive T lymphocytes and compared with that in nonproliferating CD14-positive monocytes. We then tested the activation of the same *FOXO1*-controlled genes as in the HU-treated cultured cells (see above). Although we observed predictable individual variability, all FOXO1 target genes were upregulated in proliferative CD3-positive T lymphocytes collected from all four patients (Fig. 4C). In nonproliferating CD14-positive monocytes, some genes were induced in one patient but repressed in another patient; however, the four genes were never simultaneously induced in any of the four patients (Fig. 4D), reflecting individual variability rather than induction by HU. These data reveal the activation of the FOXO1 pathway by HU treatment in proliferating cells in vivo in a physio-pathological context. Notably, these findings are consistent with a response to “low” replication stress described above.

Collectively, our data show that in cultured cells as well as in vivo, nonblocking doses of HU induce detoxification *FOXO1*-controlled gene expression in proliferating cells that should replicate their genome.

### RIR are produced by the NADPH oxidases DUOX1 and DUOX2

**Fig. 5:**
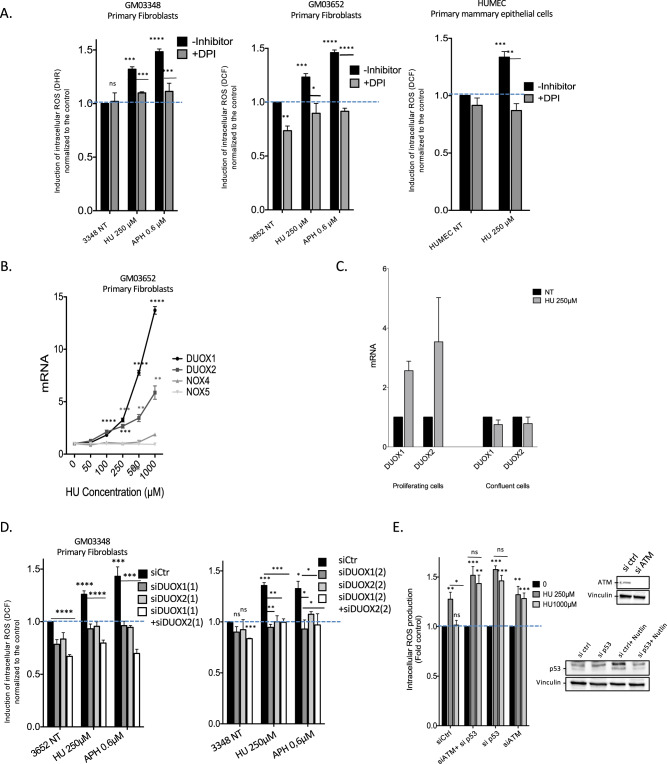
Replication stress induces DUOX1- and DUOX2-dependent RIR in primary cells. **A** Effect of DPI (an NADPH oxidase inhibitor) on RIR production in two different human primary fibroblast strains using two probes (left panels) and in one human primary epithelial cell strain (right panel). **B** Replication stress increases *DUOX1* and *DUOX2* mRNA levels in primary fibroblasts. **C** Impact of cell confluence on the induction of DUOX1 and DUOX2 mRNA levels by HU, monitored by qPCR. **D** *DUOX1* and *DUOX2* silencing impairs RIR production in primary fibroblasts. Two siRNAs for each *DUOX* were assayed: *DUOX1*(1) and *DUOX1*(2) and *DUOX2*(1) and *DUOX2*(2). **E** Impacts of *p53* or *ATM* silencing on the decrease in RIR production induced by 1 mM HU. Data from three independent experiments are presented as the mean (± SEM) level of ROS production normalized to that of the control. The efficiency of silencing is shown in the right panels. Phospho-p53 corresponds to the lower band; its level is normally very low in untreated normal cells. To detect p53, we must induce it by Nutlin (third lane). The efficiency of the siRNA was then verified (compare the third and fourth lanes).

Given that RIR appear to be a cell autonomous-regulated process, they should be tightly controlled by the cell. Thus, we then aimed to determine the cellular pathway that controls RIR production. The primary function of NADPH oxidases is the cell-regulated production of ROS [40, 41]. Remarkably, exposure of primary mammary epithelial cells or fibroblasts to diphenylene iodonium chloride (DPI), an inhibitor of all NADPH oxidases [42], abrogated RIR production (Fig. 5A). This finding suggests that RIR should be produced by one or several of the cellular enzymes NADPH oxidases.

Monitoring the mRNA expression of the seven identified NADPH oxidases revealed that *DUOX1* and *DUOX2* mRNA levels exclusively increased substantially in a HU dose-dependent manner. Neither *NOX4* nor *NOX5* mRNA levels were affected by exposure of the cells to HU (Fig. 5B). Other NADPH oxidase mRNAs were not detected. Consistent with the inhibition of RIR production, cell confluence abrogated the stimulation of *DUOX1* and *DUOX2* mRNA levels by HU (Fig. 5C).

To evaluate the involvement of *DUOX1* and/or *DUOX2* in RIR production, we silenced the expression of each gene using siRNA (siRNA efficiency is depicted in Fig. S9). Knockdown (KD) of *DUOX1* and/or *DUOX2* abolished RIR induction (Fig. 5D). Therefore, *DUOX1* and *DUOX2* produced ROS in response to HU (RIR).

### RIR produced by high doses is detoxified by ATM and p53

Remarkably, although RIR was not produced at high HU doses (1 mM, see Fig. 1), the expression of DUOX1 and DUOX2 was still induced at high HU doses (> 250 µM) in primary fibroblasts (Fig. 5B). This finding suggests that RIR are produced and should be subsequently detoxified at such high doses. ATM and p53 are prominent DDR regulators/effectors (notably double-strand breaks) that have also been described as having antioxidant functions [43, 44]. We, therefore, tested their impact on RIR levels. Although siRNA-mediated silencing of *p53* or *ATM* expression did not affect the production of RIR at 250 µM HU, it abrogated the decrease in RIR at 1 mM HU (Fig. 5E). These results were confirmed by combining chemical inhibitors against p53 and ATM (Fig. S10). These data show that RIR production itself is independent of p53 and ATM but that RIR is detoxified in a p53- and ATM-dependent manner at higher HU doses. Indeed, treatment with ATM and p53 inhibitors did not rescue DNA synthesis upon exposure to 1 mM HU (Fig. S4C), supporting that the suppression of RIR at high doses results from the detoxifying activities of p53 and ATM rather than from replication resumption.

Note that in primary fibroblasts, the activation/phosphorylation of p53 and γ-H2AX, a marker of DNA double-strand breaks (DSBs), was detectable after treatment with HU doses ranging from 250 to 1000 µM for p53 and from 500 to 1000 µM for γ-H2AX (Fig. S11). Similarly, with APH, the phosphorylation of p53 occurred for doses from 2.4 to 4.8 µM and for γ-H2AX at doses from 0.6 to 1.2 µM, with CPT activation/phosphorylation arising from 20 to 50 nM for p53 and 10 to 50 nM for γ-H2AX (Fig. S12). Remarkably, all these events occurred for doses equivalent to and higher than the doses generating the peak of RIR, which are 250 µM, 0.6 µM and 10 nM for HU, APH and CPT, respectively (compare Figs. S11, S12 with Fig. 1B, F, G). Thus, it is tempting to speculate that the accumulation and detection of DSBs might dictate the activation of the canonical DDR that, *in fine*, would lead to the detoxification of the RIR.

Collectively, these data show that replication stress is signaled by primary cells as a function of stress severity, defining the noncanonical LoL-DDR and the p53/ATM-dependent high-level stress DDR (canonical DDR).

### NF−κB controls *DUOX1* and *DUOX2* expression under replication stress and RIR production

**Fig. 6:**
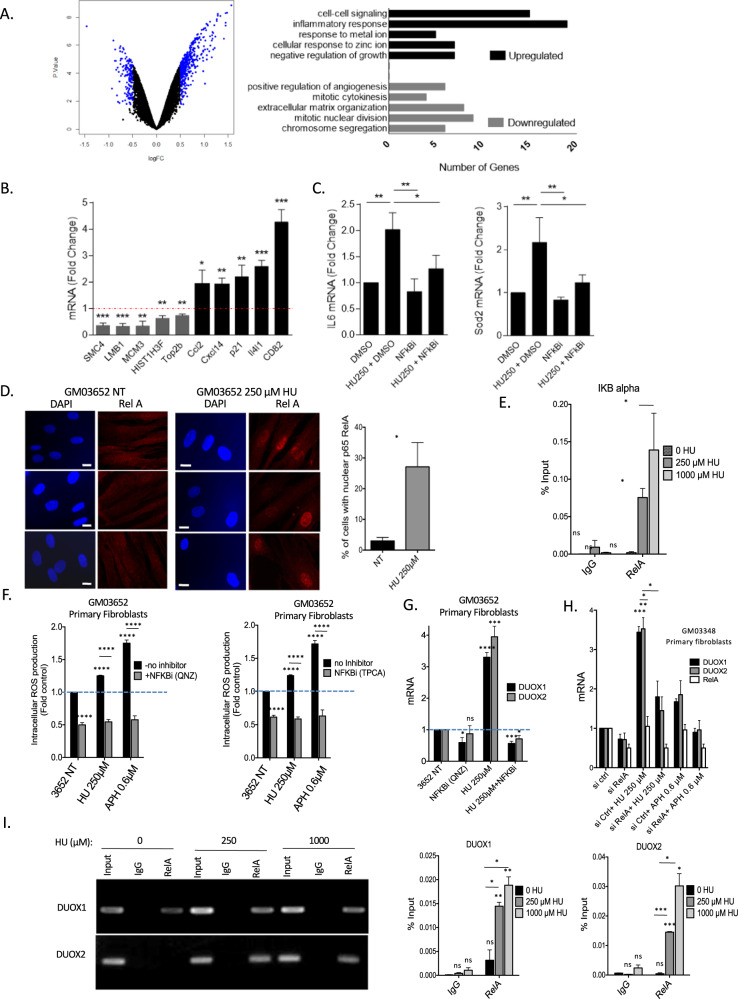
Control of RIR and *DUOX1* and *DUOX2* expression by NF-κB upon HU treatment. **A** Transcriptome analysis. Left panel: Volcano plot from microarray data comparing primary human fibroblasts (GM03348) treated (or not) with 250 µM HU. The targets with log2 (fold change - FC) > 0.5 and < −0.5 and an adjusted p value of 0.05 are highlighted in blue. Right panel: Gene Ontology analysis of down- and upregulated genes. **B** Validation of microarray analysis results by real-time RT–PCR analysis of the downregulated (SMC4, LMB1, MCM3, HIST1H3F and Top2b) and upregulated (Ccl2, Cxcl14, p21, Il4l1, and CD82) genes. **C** Real-time RT–PCR analysis of IL6 and SOD2 using cDNA generated from primary human fibroblasts (GM03348) treated (or not) with 250 µM HU and DMSO or an NF-κB inhibitor (QNZ). **D** Nuclear translocation of RelA upon HU exposure. Immunofluorescence staining for the NF-B subunit RelA (red) in primary fibroblasts. Left panel: representative photos of immunofluorescence staining for RelA (red) in primary fibroblasts. Nuclei were counterstained with DAPI (blue). Scale bars: 10 µm. Right panel: quantification of the nuclear translocation of RelA upon exposure to 250 µM HU. At least 200 cells were counted. **E** Binding of RelA to an established NF-κB target, the IκB gene promoter. **F** Inhibition of NF-κB with two different inhibitors in primary fibroblasts. **G** Impact of the inhibition of NF-κB on the mRNA expression of *DUOX1* and *DUOX2*. **H** Silencing RelA inhibits the expression of the *DUOX1* and *DUOX2* mRNAs. **I** Binding of RelA to the NF-κB RE sequences located upstream of the TSSs in the *DUOX1* and *DUOX2* genes. Top panel: electrophoretic analysis of the PCR-amplified fragments resulting from the RelA ChIP experiment. IgG: precipitate with a secondary antibody without the primary antibody. RelA: precipitation with the RelA antibody. Bottom panels: qPCR analysis and quantification relative to the input. ChIP was performed on primary GM03348 fibroblasts treated with or without HU using an anti-RelA antibody. Data from at least three independent experiments are presented (error bars,  ± SEM).

To identify pathways that regulate *DUOX1* and *DUOX2* expression and consequently RIR upon “low” (nonblocking) replication stress, we performed a microarray analysis comparing nontreated *versus* 250 µM HU-treated primary human fibroblasts (Fig. 6A); this HU dose corresponds to generating the peak of RIR (see Fig. 1B). Using cutoff values of log2 (fold change - FC) > 0.5 and < −0.5 and an adjusted *p*-value of 0.05, we identified 152 down- and 416 upregulated genes in HU-treated cells (Fig. 6A and Tables S13A1, S13A2, and S13A3). Gene Ontology analysis revealed the downregulation of cell cycle-regulating genes, whereas genes involved in inflammation, negative regulation of growth, metabolism of metal and zinc ions, and cell–cell signaling were upregulated (Fig. 6A). This pattern is consistent with the known impact of HU on the cellular response to replication stress and on cell division. Remarkably, 69 genes with increased expression upon 250 µM HU treatment harbored binding sites for p65 (RelA), a member of the NF-κB signaling pathway, in their promoters (Fig. S13B). Gene expression data were then validated by real-time RT–PCR analysis of up- and downregulated targets (Fig. 6B). Then, we verified that two classic NF-κB targets (IL6 and SOD2) detected in our microarray analysis were actually upregulated in an NF-κB-dependent manner after exposure to 250 µM HU (Fig. 6C).

Moreover, gene set enrichment analysis (GSEA) of RNA sequencing indicated the upregulation of the NF-κB pathway in vivo in CD3-positive T cells (proliferative) in peripheral blood samples from the four CMML patients chronically treated with HU (Fig. S13C).

Consistently, the NF-κB subunit RelA accumulates in the nucleus following exposure to 250 µM HU (Fig. 6D), and chromatin immunoprecipitation (ChIP) showed that HU stimulated the binding of RelA to the promoter of the *IκB* gene, a known target of NF-κB (Fig. 6E). Collectively, these data show that 250 µM HU activates the NF-κB pathway, leading to the expression of NF-κB-dependent genes.

NF-κB is involved in a variety of physiological and pathological pathways, including cell proliferation and death, immune and inflammatory responses, and tumor immunosurveillance [45]. NF-κB can be induced by genotoxic stresses, including strong replication stress [46, 47]. Here, we show that this pathway is also activated by nonblocking replication stress. In silico analysis revealed a RelA/p65 binding site upstream of the transcription start sites of both the *DUOX1* and *DUOX2* genes but not in the other NADPH oxidase-encoding genes (http://www.genecards.org/). To test whether NF-κB is involved in RIR production through the upregulation of *DUOX1* and *DUOX2* expression, we inhibited NF-κB with chemical inhibitors. Treatment of the cells with NF-κB inhibitors suppressed RIR production induced by either HU or APH (Fig. 6F). Moreover, the NF-κB inhibitors also abrogated the induction of *DUOX1* and *DUOX2* mRNA by HU (Fig. 6G). More specifically, *RELA* silencing suppressed the HU-increased levels of both *DUOX1* and *DUOX2* mRNAs (Fig. 6H). Finally, ChIP experiments also revealed i) that RelA binds to NF-κB-responsive regions of both the *DUOX1* and *DUOX2* promoters and ii) that this binding is stimulated by exposure of the cells to HU (Fig. 6I).

These data reveal that NF-κB controls RIR production through *DUOX1* and *DUOX2* gene expression.

### PARP1 controls the production of RIR

**Fig. 7:**
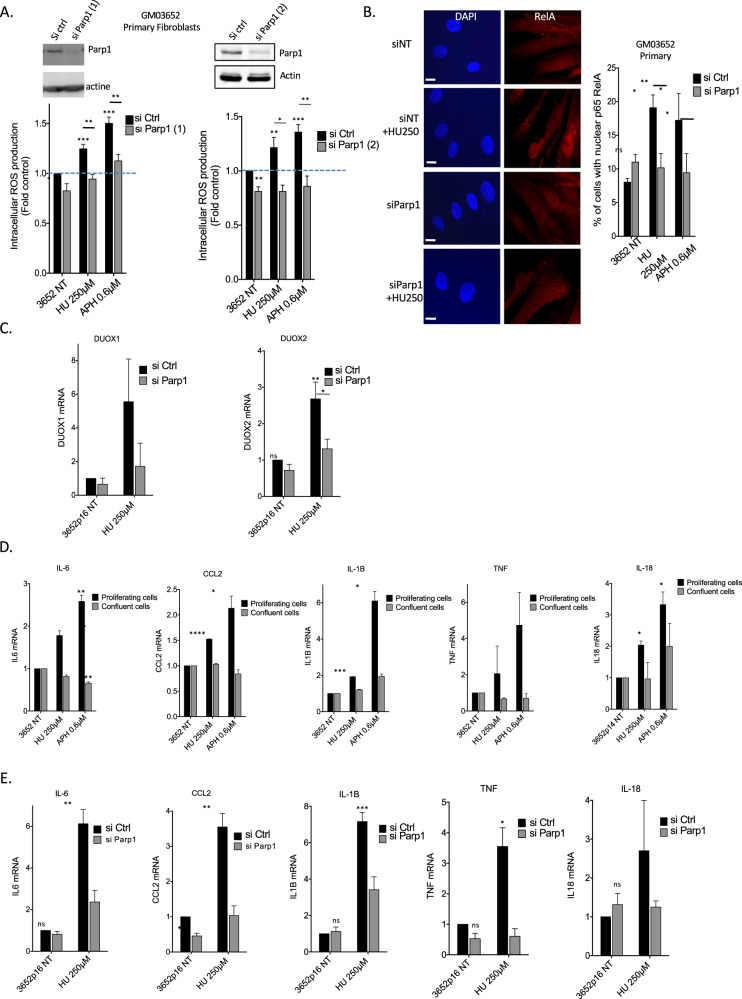
PARP1 controls RIR and cytokines production. **A** Silencing *PARP1* (2 different siRNAs) abolished the induction of HU- or APH-induced RIR. Upper panels: immunoblots of *PARP1* silencing in primary fibroblasts. Lower panels: quantification of ROS (DCFA). **B** Silencing *PARP1* inhibits the HU-induced translocation of RelA in primary fibroblasts. Left panel: representative photos of immunofluorescence staining for RelA (red) in primary fibroblasts. Nuclei were counterstained with DAPI (blue). Scale bars: 10 µm. Right panel: quantification of cells with nuclear RelA. Right panel: quantification of the nuclear translocation of RelA. At least 200 cells were counted. The data from four independent experiments are presented. **C** Impact of *PARP1* silencing on the expression of *DUOX1* and *DUOX2* (RT-qPCR). **D** Impact of proliferation *versus* confluence on the expression of 5 classic cytokine genes upon exposure to HU (250 µM) or APH (0.6 µM) (RT–qPCR). **E** Impact of *PARP1* silencing on the expression of 5 classic cytokine genes upon exposure to HU (250 µM) (RT–qPCR).

PARP1 is involved in the response to DNA damage and can also activate NF-κB via a mechanism that does not require PARP1 enzyme activity [48–51]. PARP1 is therefore a candidate to regulate NF-κB-mediated RIR production through the upregulation of *DUOX1* and *DUOX2*. To test this hypothesis, we silenced *PARP1* expression using siRNA KD. Silencing PARP1 resulted in the loss of RIR induced by HU and APH (Fig. 7A). In contrast, PARP enzyme inhibitors did not affect RIR induction (Fig. S14).

Silencing *PARP1* prevented the nuclear accumulation of RelA (Fig. 7B) and the upregulation of *DUOX1* and *DUOX2* gene expression in response to HU or APH exposure (Fig. 7C). Thus, RIR production is controlled by PARP1, which activates the RelA-dependent NF-κB pathway to upregulate *DUOX1* and *DUOX2* gene expression.

### PARP1 controls replication stress-induced cytokine gene expression

Replication stress induces the production of inflammatory cytokines (for review, see [52, 53]). This constitutes an additional level of protection against the proliferation of cells bearing damaged genomes that acts in parallel to the DDR. Given that NF-κB directly activates the expression of inflammatory cytokine genes [52, 53], we addressed the question of whether PARP1 also controls the induction of inflammatory cytokine genes upon “low” replication stress in primary human fibroblasts. We analyzed the expression of 5 classic cytokine genes. First, we showed that 250 µM HU or 0.6 µM APH promoted cytokine gene expression in proliferating primary human fibroblasts but not in confluent cells (Fig. 7D). This finding confirms that even at such a low stress level, the induction of cytokine genes is actually correlated with the proliferation status and thus with replication stress. More specifically, silencing *PARP1* abolished the induction of cytokine gene expression upon exposure to 250 µM HU (Fig. 7E).

Collectively, these data show that the specific response to “low” (nonblocking) replication stress is controlled by the PARP1/NF-κB axis. This pathway simultaneously activates in parallel i) the RIR response (through the expression of *DUOX1* and *DUOX2*) and ii) the expression of inflammatory cytokines.

## Discussion

Here, we show that primary cells react to genotoxic stress as a function of stress intensity. At stress levels that poorly affect cell cycle distribution and DNA synthesis, our data reveal a specific noncanonical response that is thus dedicated to low-level replication stress (LoL-DDR). Because cells are challenged daily by low-level/endogenous stresses, LoL-DDR likely plays a prime role in genome integrity maintenance. LoL-DDR favors genome stability maintenance through an adaptive response. Indeed, LoL-DDR induces and controls the production of ROS (RIR), which activates the FOXO1 detoxifying pathway. Of note, the production of RIR is p53- and ATM-independent, thus corresponding to a noncanonical response to genotoxic stress. LoL-DDR is under the control of the PARP1-NF-κB axis, which in parallel also directly induces the expression of inflammatory cytokines.

RIR is induced by different agents that all generate replication stress, such as HU, APH and CPT. Moreover, HU-induced ROS production was abrogated when primary cell confluence was maintained, where DNA replication was abrogated. Collectively, these data support the concept that the ROS monitored here are induced in DNA replicating cells by a low level (nonblocking) of replication stress, thus corresponding to RIR. Consistently, the level of RIR induction appears to be low in primary fibroblasts, in which the frequency of cells in S phase is low. Nevertheless, the RIR level was comparable to that of ROS generated by exposure to 25 µM H_2_O_2_. In addition, the induction of RIR was statistically significant and highly reproducible at the same dose in each individual replicate of the experiment with each of the four different strains of primary fibroblast strains. RIR was also recorded with the same dose‒response peak curves in primary mammary epithelial cells, i.e., in another tissue. Moreover, RIRs were detected with different fluorescent probes and were confirmed using an engineered GFP. Note that the different replication stress inducers (HU, APH and CPT) induced RIR to the same extent and with similar peak-shaped curves. Remarkably, this level of ROS induction was sufficient to protect the genome from the accumulation of premutagenic lesions, such as 8-oxoG. RIRs are tightly controlled by primary cells via the PARP1/NF-κB/DUOX1/2/FOXO1 axis. Finally, HU-induced genes were also detected in vivo in a physio-pathological context. Collectively, these data attest to the existence of moderate but actual RIR production in primary cells as a cellular autonomous response.

**Fig. 8:**
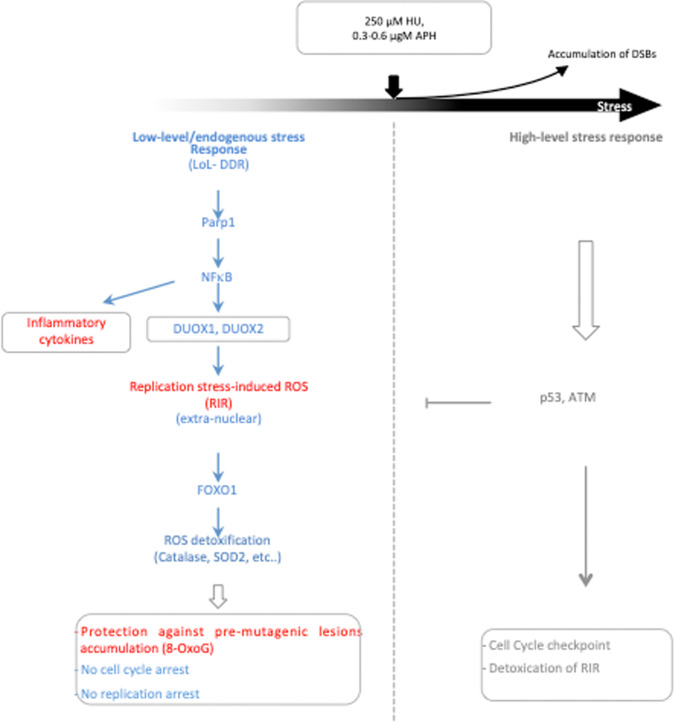
The biphasic model response to DNA damage. Primary cells adapt their response to replication stress intensity according to distinct phases: the low-level/endogenous stress response and the high-level stress response. Below a certain stress intensity threshold, cells engage the low-level response (LoL-DDR), which does not repress DNA synthesis and cell progression. The LoL-DDR response regulates the production of extranuclear ROS (RIR) under the control of cellular PARP1, NF-κB, DUOX1 and DUOX2. In parallel, NF-κB induces the expression of inflammatory cytokine genes. RIR induces the FOXO1 detoxifying program, protecting against the accumulation of premutagenic lesions, such as 8-oxoG, in an adaptive-like detoxification response. Above the threshold, cells accumulate DSBs and activate the canonical DDR, which detoxifies RIR.

Our data (summarized in Fig. 8) support a biphasic model for cellular responses to replication stress in primary cells (Fig. 8). At low-level stress (equivalent to ≤ 250 µM HU, ≤ 0.6 µM APH or ≤ 10 nM CPT), LoL-DDR generates RIR, which are extranuclear and thus do not jeopardize genome integrity. Our data dissect and characterize cell control of RIR production: RIRs are synthetized by the cellular NADPH oxidases DUOX1 and DUOX*2* under the control of NF-κB, which is activated by the PARP1 protein. Note that the presence of the PARP1 protein, but not its activity, is required for NF-κB activation, as previously shown in other systems [50, 51]. RIR protects the genome from the accumulation of premutagenic oxidative lesions, such as 8-oxoG (Fig. 8). Given the genotoxic potential of ROS, producing ROS to protect genome integrity seems paradoxical. However, several mechanisms contribute to the prevention of the potential deleterious consequences of RIR. First, RIR are excluded from the nucleus. Second, the level of RIR in primary fibroblasts is moderate, as discussed above. Third, RIR triggers a FOXO1-mediated ROS detoxification program, resulting in an adaptive response. Noteworthy, the RIR is responsible for FOXO1-dependent expression of a suite of ROS detoxifying enzymes, at least one of which (SEPP1) is nuclear localized, which might account for the absence of nuclear ROS. The level of RIR should correspond to an equilibrium because the FOXO1-detoxifying pathway induced by RIR should detoxify RIR, maintaining a low and controlled level of ROS. Note that the RIR-induced detoxification program also protects against other sources of ROS, such as exogenous stress, as shown upon H_2_O_2_ exposure. Replication stress also induces mitochondrial ROS, which are potentially detrimental to genome stability. The fact that the RIR-induced detoxification program protects against exogenous ROS suggests that it should also be able to protect against other endogenous sources of ROS, such as mitochondrial ROS. This hypothesis is supported by the decrease in 8-oxoG accumulation upon nonblocking replication stress.

In a similar way, NADPH oxidases trigger redox signaling that favors resistance to oxidative stress and promotes longevity in worms [54], thus supporting our interpretations. In humans, various NADPH oxidases are often upregulated in cancers. In contrast, only DUOX1 and DUOX2 are lost in different types of tumors [55], highlighting the protective effects of DUOX1- and DUOX2-controlled ROS. Moreover, antioxidants (including NAC, which abrogates RIR), which have been proposed to protect against carcinogenesis, in fact foster lung carcinomas and metastasis [56–58]. This argues for potential beneficial roles for ROS in the homeostasis of human cells. More generally, defects in each of the players of the LoL-DDR response described here (NF-κB, PARP1, FOXO1, DUOX1 and DUOX2) share common phenotypes, such as defects in cell homeostasis and metabolism, aging and cancer predisposition [55, 59–64]. Of note, LoL-DDR is misregulated in transformed cell lines (data to be published).

In addition to RIR production, NF-κB activation by low replication stress induced the production of inflammatory cytokines (Figs. 6A–C, 7D, E and 8) that might activate innate immunity. This constitutes a potential additional level of protection against damaged DNA. Indeed, by eliminating damaged cells, innate immunity participates in the maintenance of genome stability. Replication stress induces the production of inflammatory cytokines through the cGAS-STING pathway, which activates NF-κB (for review, see [52, 53]). Noncanonical activation of STING by ATM has been reported to mediate NF-κB signaling [65]. Here, our data reveal an additional level of molecular regulation of this process, showing that the the production of inflammatory cytokines can be directly activated through, i.e., the PARP1/NF-κB axis. The PARP1/NF-κB axis has been described as a proinflammatory pathway in several other situations (for example, in macrophages during *Trypanosoma cruzi* infection and Chagas disease) [66]. Here, we show its involvement in the induction of inflammatory cytokine genes upon “nonblocking“ replication stress in human primary fibroblasts. This finding supports a proinflammatory role in the response to such DNA stress, even at low levels. Therefore, unraveling the potential intricacy between RIR, NF-κB and the STING pathway for the production of inflammatory cytokines and innate immunity induction in response to “low” stress represents an exciting challenge for future studies.

Because ROS represent a threat to the cell, RIR should be tightly controlled. Here, we demonstrate that cells tightly control RIR production through the PARP1/NF-κB pathway, which regulates the expression of the *DUOX1* and *DUOX2* genes. Increasing ROS levels with stress severity might ultimately jeopardize DNA and other cellular components. Therefore, when the replication stress intensity reaches a certain threshold, DUOX1 and 2 are still activated, but RIR are detoxified by p53 and ATM, which also control the canonical DDR (Fig. 8). Note that the decrease in RIR (and presumably the canonical DDR activation) starts when DSBs accumulate. Hence, LoL-DDR could be considered a “precanonical DDR” response, which authorizes cells to replicate their DNA but induces the FOXO1 detoxifying program through the production of RIR, preventing the accumulation of premutagenic lesions, such as 8-oxoG. Moreover, given that ROS can alter DNA integrity, the exclusion of RIR from the nuclei of primary cells limits these risks.

We propose that the LoL-DDR pathway helps cells cope with low-level replication stress. By reducing the level of endogenous damage, LoL-DDR allows the management of the need for common resources (such as nucleotides) for both DNA replication and repair. However, at high replication stress, RIR becomes superfluous and even potentially dangerous. Thus, RIRs are inactivated by ATM and p53, and the canonical DRR is activated, leading to cell cycle arrest.

Based on the importance of replication stress in senescence and cancer initiation, efficient and suitable responses to stress must be tightly adjusted. The data presented here highlight a fine-tuned cellular response to stress. Specifically, the cell has the capacity to precisely adapt its response to stress severity. In particular, the pathway we have identified and characterized likely plays an essential role in genome stability maintenance against low-level/endogenous stresses. This issue is particularly important because cells are chronically exposed to low-level/endogenous stresses throughout their lifespan in contrast to acute exposure to severe stress. Therefore, the precise and appropriate regulation of these very sensitive processes is essential to protect the genome against these daily pernicious and inevitable threats.

## Methods and Material

### Cell culture and treatments

Cells were grown at 37 °C with 5% CO_2_ in modified Eagle’s medium (MEM). Primary human skin fibroblasts were grown in MEM (Gibco, Life Technologies) supplemented with 20% fetal calf serum (FCS; Lonza Group, Ltd.). Primary human mammary epithelial cells (HuMECs) were provided by Thermo Fisher Scientific and cultured according to the manufacturer’s recommendations. Primary fibroblasts were exposed to HU, APH or CPT for 72 h at 37 °C. For antioxidant treatment, primary fibroblasts were exposed to 2 mM NAC (Sigma‒Aldrich, St. Louis, MO, USA) for 72 h. For the inhibition of *p53, ATM*, or *PARP1*, primary fibroblasts were transfected with a *p53*-targeted siRNA (siTP53, si On-Target Plus SMARTpool L-003329-00), an *ATM*-targeted siRNA (siATM, si On-Target Plus SMARTpool L-003201-00) or a control nontargeting siRNA (si On-target Plus nontargeting pool D-001810-10-05), all of which were purchased from Dharmacon Inc. (Lafayette, CO), or a *PARP1*-targeted siRNA (siPARP1, Santa Cruz Biotechnology or siParp1 2^nd^ siRNA, Ambion) using Interferin transfection reagent (Invitrogen) according to the manufacturer’s protocol. For the inhibition of *FOXO1*, primary fibroblasts were transfected with a control nontargeting siRNA purchased from Santa Cruz Biotechnology or a *FOXO1*-targeted siRNA (siFOXO1(1) GAGCGUGCCCUACUUCAAGGA or siFOXO1(2) GUUAAGUUCUGGGCUCGCGCdTdT) using the Amaxa™ Basic Nucleofector™ Kit for Primary Mammalian Fibroblasts (Lonza) according to the manufacturer’s protocol. To silence *DUOX1* and *DUOX2*, *DUOX1*-targeted siRNA (siDUOX1(1), siOn-Target Plus SMARTpool L-008126-00) purchased from Dharmacon, Inc. (Lafayette, CO) or siDUOX1(2) (GCUAUGCAGAUGGCGUGUA)TT and a *DUOX2*-targeted siRNA siDUOX2(1) purchased from Santa Cruz Biotechnology or siDUOX2(2) (CGCAGUCAAUGUCUACAUCTT) were used. For the inhibition of NF-kB, fibroblasts were treated with 1 µM QNZ (EVP4593) or 20 µM TPCA-1 (Santa Cruz and Selleckchem, respectively) for 72 h. The RelA-targeted siRNA and control nontargeting siRNA were purchased from Santa Cruz Biotechnology. For the activation of p53, fibroblasts were treated with 2 µM Nutlin-3 (an MDM2 inhibitor IV, Calbiochem).

### Measurement of cellular ROS production by FACS analysis

Cellular ROS production was measured using a CM-H2DCFDA (2’,7’-dichlorofluorescein diacetate) (Life Technologies, USA) or dihydrorhodamine 123 (DHR 123) (Sigma) assay kit according to the manufacturer’s protocol. Approximately 10^5^ cells/well were plated into 6-well plates and incubated at 37 °C with 5% CO_2_. After 3 days, the cells were rinsed with PBS and incubated with 10 µM CM-H2DCFDA or DHR 123 in DMEM supplemented with 1% FBS for 45 min at 37 °C in the dark. The cells were trypsinized and resuspended in DMEM supplemented with 1% FBS. The pelleted cells were washed again, and the live pelleted cells were resuspended in PBS and analyzed on a BD Accuri C6 flow cytometer (BD Biosciences, San Diego, CA) equipped with an FL1 laser (515–545 nm). The data are presented as the mean percentages of four independent experiments.

### Measurement of cellular ROS production using the green fluorescent protein RoGFP

RoGFP was expressed in primary fibroblasts using modified pEGFP-N1 (RRID:Addgene_38120) as the expression vector and JetPei as the transfection reagent. After the cells were incubated in culture medium treated with or without HU or APH for 72 h at 37 °C, the cells were washed twice with Hanks’ balanced salt solution. For pEGFP-N1/roGFP1, the cells were imaged on a *Zeiss* Observer Z1 microscope with a Hamamatsu ORCA Flash 4*LT* camera. Images were acquired using MetaMorph software (Molecular Devices). For dual excitation ratio imaging, excitation filters at wavelengths of 400 nm and 488 nm were used, and an emission filter at a wavelength of 535 nm was used. The fluorescence excitation ratio was obtained by dividing the intensities of the cells using excitation filters at 400 nm and 488 nm.

For pEGFP-N1/roGFP-NLS (nuclear localization), the cells were incubated with DAPI (1 μg/ml) and imaged using a microscope. The images were captured using the 63x oil immersion objective of a motorized Axio Imager Z2 epifluorescence microscope (Carl Zeiss) equipped with a high-sensitivity cooled interline CCD camera (Cool SNAP HQ2; Roper Scientific) and a PIEZO stage (Physik Instrumente). Images were acquired using MetaMorph software (Molecular Devices). In each case, 300–500 cells were analyzed per condition.

### Western blot analysis

Cells were suspended in lysis buffer (8 M urea, 1 M thiourea, 4.8% CHAPS, 50 mM DTT, 24 mM spermine dehydrate, protease inhibitor cocktail (Complete Lysis Buffer; Roche, Meylan, France), and 0.1 mM Na_3_VO_4_), and proteins were extracted after repeated mechanical disruption of the lysate through a needle attached to a 0.3 ml syringe. After the samples were incubated for 1 h at room temperature, they were cleared by centrifugation at 13,000 rpm. For each blot, equal amounts (30 mg) of protein from each sample were loaded onto the gel. Electrophoretic separation, transfer to a nitrocellulose membrane and antibody probing were performed using standard techniques. The proteins were visualized using the ECL Western blotting System. Actin was probed with a 1:1000 dilution of a specific antibody (Sigma‒Aldrich), and the nonphosphorylated and phosphorylated forms of Chk1 were detected using a 1:500 dilution of an anti-P (S317)-Chk1 antibody (Cell Signaling Technology). A 1:500 dilution of anti-phospho-histone H2A. X (Ser139) antibody (Thermo Fisher Scientific Cat # MA5-31471, RRID: AB_2787103) was used to detect phosphorylated histone H2A.X levels, and phosphorylated p53 was detected using an anti-P (S15)-p53 antibody (Aeonian Biotech Cat # AE00218, RRID: AB_2813802). OGG1 and p53 were probed with a specific antibody at a 1:1000 dilution (Novus Biologicals). MTH1 was probed with a 1:500 dilution of a specific antibody (Invitrogen). FOXO1 was probed with a 1:500 dilution of a specific antibody (Cell Signaling), and PARP1 or p21 was probed with a 1:500 dilution of a specific antibody (Santa Cruz Biotechnology).

### Western blot analysis of ATM

Protein lysates were mixed with Laemli containing DTT and heated at 70 °C for 10 min. Samples were then separated on a NuPAGE 3–8% tris-acetate protein gel (Thermo Fisher Scientific) at 150 V for 80 min with 1x NuPAGE Tris acetate SDS running buffer containing NuPAGE antioxidant (Thermo Fisher Scientific) during electrophoresis. The fractionated proteins were transferred to a nitrocellulose membrane with 1x NuPAGE Tris acetate transfer buffer containing 15% ethanol. The membrane was blocked with 5% milk in PBS containing 0.1% Tween 20 and then incubated overnight at 4 °C with an ATM antibody (Santa Cruz). The proteins were visualized using the ECL Western blotting System.

### 8-OxoG measurement

Genomic DNA was extracted and enzymatically digested using an optimized protocol that minimizes DNA oxidation during the procedure [67]. Then, 8-oxoG levels were quantified using isotope dilution high-performance liquid chromatography coupled with electrospray ionization tandem mass spectrometry (HPLC–MS/MS) as previously described [36]; ^15^N_5_-8-oxoG served as the internal standard. In addition to the mass spectrometric detector, the system was equipped with a UV detector that was set at 260 nm to measure the quantity of normal nucleosides. The results are expressed as the number of 8-oxoG per million normal nucleosides.

### Immunofluorescence

The cells were grown on glass coverslips, fixed with 2% paraformaldehyde and permeabilized with 0.5% Triton X-100. After blocking with PBS containing 3% BSA and 0.05% Tween 20, the cells were incubated with an anti-RelA primary antibody (Santa Cruz Biotechnology Cat# sc-372, RRID: AB_632037) diluted in PBS containing 3% BSA and 0.05% Tween 20. After washing with PBS containing 0.05% Tween 20, the cells were incubated with an Alexa Fluor 568-conjugated anti-mouse secondary antibody (Invitrogen, Molecular Probes) and stained with DAPI. For 8-oxoG detection in nuclear DNA, the cells were grown on glass coverslips, fixed with 2% paraformaldehyde and permeabilized with 0.5% Triton X-100. Then, the cells were denatured with 2 N HCl to allow access of the chromatin to the antibody. The cells were washed three times in PBS and neutralized with 50 mM Tris–HCl (pH 8.8) before blocking with 2% fetal calf serum in PBS containing 0.05% Tween 20. The cells were incubated with a mouse anti-8-oxo-dG antibody (clone N45.1, 1:100, ab48508 Abcam). After washing with PBS containing 0.05% Tween 20, a goat anti-mouse IgG Alexa 568 (Invitrogen) secondary antibody was used. Nuclear DNA was counterstained with DAPI (Dako). Images were captured using a Zeiss motorized Axio Imager Z2 epifluorescence microscope with a 63x/1.4 NA oil immersion objective equipped with a Hamamatsu camera. Data were acquired using AxioVision (AxioVision Imaging System, RRID: SCR_002677).

### Cell cycle analysis and BrdU incorporation

Cells were incubated in culture medium treated with or without HU for 72 h at 37 °C, and 5-bromo-2-deoxyuridine (BrdU, Sigma) was added to the culture media at a final concentration of 10 µM for 30 min. Pelleted cells were detached with trypsin, fixed with 80% ethanol, and resuspended in 30 mM HCl/0.5 mg/ml pepsin. BrdU was immunofluorescently labeled with a mouse anti-BrdU antibody (DAKO, clone Bu20a) and a fluorescein-conjugated donkey anti-mouse antibody (Life Technologies), and the cells were stained with propidium iodide (PI; 25 µg/ml) in the presence of ribonuclease A (50 µg/ml). Flow cytometry analyses were performed using an Accuri C6 flow cytometer (BD Biosciences).

### RNA extraction and quantitative RT–PCR (TaqMan)

Total RNA was isolated using a Macherel-Nagel NucleoSpin RNA Kit according to the manufacturer’s instructions. cDNAs were generated from 2 μg of total RNA using random hexamers and RevertAid Premium Reverse Transcriptase (Thermo Fisher Scientific). The following primers were used for the TaqMan® probe-based qPCR assay (Applied Biosystems): DUOX1 (Hs00213694_m1), DUOX2 (Hs00204187_m1), NOX4 (Hs00276431_m1), NOX5 (Hs00225846_m1), *RELA* (Hs00153294_m1), *SEPP1* (Hs01032845_m1), *CATALASE* (Hs00156308_m1), GPX1 (Hs00829989_gH), SOD2 (Hs00167309_m1), TXNRD1 (Hs00917067_m1), *NQO1* (Hs01045993_g1), *FTL* (Hs00830226_gH), *MGST1* (Hs00220393_m1) *DUOX1* (Hs00213694_m1), and *DUOX2* (Hs00204187_m1). Beta-*ACTIN* (Hs99999903_m1) served as the internal control. The sequences of the primers used for the SYBR assays are shown in Supplemental Data Table S15. Quantitative RT–PCR was performed using the Applied Biosystems 7300 Real-Time PCR System. All experiments were performed in triplicate.

### ChIP and quantitative PCR (ChIP‒qPCR)

Primary fibroblasts were treated with or without 250 µM or 1000 µM HU, cross-linked with 1% formaldehyde for 10 min, and then incubated with 125 mM glycine for 5 min. Cells were washed with ice-cold PBS, collected by scraping and centrifuged at 1000 x g for 5 min at 4 °C. The supernatant was removed, and the pellets were resuspended in lysis buffer (50 mM HEPES (pH 8.0), 140 mM NaCl, 1 mM EDTA, 1% Triton X-100, 0.1% sodium deoxycholate, 0.5% SDS, and freshly added protease inhibitors) and incubated on ice for 10 min before sonication. Sonicated chromatin was diluted in radioimmunoprecipitation assay (RIPA) buffer (50 mM Tris (pH 8.0), 1% Triton X-100, 1 mM EDTA, 150 mM NaCl, 0.1% sodium deoxycholate, and 0.05% SDS, freshly added protease inhibitors) and incubated with pretreated beads and 5 µg of the RelA antibody (Santa Cruz Biotechnology Cat# sc-372, RRID: AB_632037) overnight at 4 °C. The beads were then washed with washing buffer 1 (20 mM Tris-HCl (pH 8.0), 1% Triton X-100, 2 mM EDTA, 150 mM NaCl, 0.1% SDS, and freshly added protease inhibitors) for 5 min followed by sequential washes with buffer 2 (20 mM Tris-HCl (pH 8.0), 1% Triton X-100, 2 mM EDTA, 300 mM NaCl, 0.1% SDS, and freshly added protease inhibitors), buffer 3 (10 mM Tris-HCl (pH 8.0), 250 mM LiCl, 1% NP40, 1 mM EDTA, 1% sodium deoxycholate, and freshly added protease inhibitors) and TE buffer (10 mM Tris-HCl (pH 8.0) and 1 mM EDTA; two times) for 5 min each. One hundred microliters of elution buffer (10 mM Tris-HCl (pH 8.0) and 1 mM EDTA) was added, and the beads were incubated with RNase A (400 μg/ml) and NaCl (600 mM) in a Thermo mixer for 1 h at 37 °C at 1400 rpm. Then, proteinase K (400 µg/ml) and 1% SDS were added, and the mixture was incubated at 65 °C overnight with agitation. The beads were precipitated, and the supernatants were treated with phenol/chloroform/isoamyl alcohol followed by centrifugation at 13,000 x g for 5 min. The supernatants (aqueous phase) were incubated with 300 mM sodium acetate and cold ethanol for 1 h at −80 °C followed by centrifugation at 13,000 x g for 30 min. The DNA pellets were washed with 70% ethanol and resuspended in nuclease-free water. The DNA was subjected to qPCR identification with PowerUp SYBR Green Master Mix (Applied Biosystems) using the Applied Biosystems 7300 Real-Time PCR System. The data were analyzed with a standard curve-based method. The reference qPCR primers were as follows:

Human DUOX1, GPH1004464(-)02 A (Qiagen) and

Human DUOX2, GPH1018346(-)08 A (Qiagen).

### Microarray

Gene expression analysis was performed with an Agilent® SurePrint G3 Human GE 8x60K Microarray (Agilent Technologies, AMADID 39494) with the following dual-color design. The test samples were labeled with Cy5, whereas the control samples were labeled with Cy3 using a two-color Agilent labeling kit (Low Input Quick Amp Labeling Kit 5190-2306) adapted for a small amount of total RNA (100 ng total RNA per reaction). Hybridization was then performed on the microarray using 825 ng of each linearly amplified cRNA-labeled Cy3 or Cy5 sample following the manufacturer’s protocol (Agilent SureHyb Chamber; 1650 ng of labeled extract; duration of hybridization: 17 h; 40 µL per array; temperature: 65 °C). After washing in acetonitrile, slides were scanned using an Agilent G2565 C DNA microarray scanner under default parameters (100° PMT, 3 µm resolution, 20 °C in a free ozone concentration environment). Microarray images were analyzed by using Feature Extraction software version (10.7.3.1) from Agilent Technologies. Default settings were used.

### Microarray data processing and analysis

Raw data files from Feature Extraction were imported into LIMMA (RRID:SCR_010943) (Smyth, 2004, Statistical Applications in Genetics and Molecular Biology, vol 3, No 1, article 3), an R package from the Bioconductor project (Bioconductor, RRID:SCR_006442), and processed as follows: gMedianSignal and rMedianSignal data were imported, control probes were systematically removed, and flagged probes (gIsSaturated, gIsFeatpopnOL, gIsFeatNonUnifOL, rIsSaturated, rIsFeatpopnOL, and rIsFeatNonUnifOL) were set to NA. Intraarray normalization was performed by loess normalization followed by quantile normalization of both the Cy3 and Cy5 channels. Then, interarray normalization was performed by quantile normalization on M values. To obtain a single value for each transcript, the mean of each replicated probe was used to summarize the data. Missing values were inferred using the KNN algorithm from the package ‘impute’ from R Bioconductor.

Normalized data were then analyzed. To identify differentially expressed genes between the two groups, we started by fitting the data to a linear model. Then, we used an empirical Bayes method to moderate the standard errors of the estimated log-fold changes. The top-ranked genes were selected based on the following criteria: an absolute fold-change > 2 and an adjusted p value (FDR) < 0.05. Microarray Gene Ontology analysis and transcription factor-binding site predictions were performed using the DAVID (DAVID, RRID:SCR_001881) platform [68, 69].

### Whole-transcriptome RNA-seq

RNA integrity (RNA integrity number ≥ 7.0) was assessed on an Agilent 2100 Bioanalyzer (Agilent), and RNA quantity was determined using Qubit (Invitrogen). The SureSelect Automated Strand Specific RNA Library Preparation Kit was used according to the manufacturer’s instructions with the Bravo Platform. Briefly, 150 ng of total RNA was used for poly-A mRNA selection using oligo(dT) beads and subjected to thermal mRNA fragmentation. The fragmented mRNA samples were subjected to cDNA synthesis and further converted into double-stranded DNA using the reagents supplied in the kit, and the resulting dsDNA was used for library preparation. The final libraries were bar-coded, purified, pooled together in equal concentrations and subjected to paired-end sequencing on a NovaSeq-6000 sequencer (Illumina) at Gustave Roussy.

### RNA-seq analysis

QC controls were performed using fastqc v0.11.7. For each sample, the number of sequenced fragments was assigned to each gene/transcript using Salmon (RRID:SCR_017036) 13.0.1 [https://salmon.readthedocs.io/en/latest/salmon.html]. We first built the quasimapping-based index using an auxiliary k-mer hash over k-mers of length 31, retaining sequence-identical duplicate transcripts (duplicate transcripts that appear in the input were retained and quantified separately) on the nucleotide sequences of all transcripts on the reference chromosomes of genecode v27. Quantification was performed at both the gene and transcript levels using comprehensive gene annotation on the reference chromosomes (GTF) from genecode v27 through the option ‘geneMap’ with numBoostraps set to 100 and libType set to ‘A’, enabling gcBias, seqBias (RRID:SCR_006832) and validateMappings. We used DESeq2 (RRID:SCR_000154) to perform differential analysis at the gene level [70]. To perform differential analysis at the transcript level, Salmon’s data output was first prepared for Sleuth v0.29 [71] using Wasabi v0.2 (https://github.com/COMBINE-lab/wasabi). Pathway enrichment analysis of RNA-seq data was performed via gene set enrichment analysis (GSEA) of NF-kB targets (the list of genes was obtained from HINATA_NF-kB_targets_keratinocyte_up). Genes with at least a 2-fold increase were considered upregulated.

### Patients with chronic myelomonocytic leukemia (CMML) treated with HU

Peripheral blood samples were collected into EDTA-coated tubes at inclusion and after HU treatment from 5 patients with a CMML diagnosis according to the 2016 World Health Organization criteria. Mononuclear cells were isolated from blood samples using density gradient centrifugation with Pancoll (Pan-Biotech, Dutscher, Brumath, France), and CD3^+^ lymphocytes and CD14^+^ monocytes were sorted with magnetic beads and the AutoMacs system (Miltenyi Biotech, Paris, France). Total RNA from patient T lymphocytes (CD3^+^ cells) and monocytes (CD14^+^ cells) was isolated using an RNA/DNA/Protein Purification Plus Kit (Norgen Biotek Corp.). Reverse transcription was performed using random hexamers and RevertAid Premium Reverse Transcriptase (Thermo Fisher Scientific), and quantitative real-time PCR was performed.

Blood samples from CMML patients with informed consent at inclusion and after 6 cycles of HU have been used (NCT02214407) based on a collaboration with the Groupe Francophone des Myélodyplasies (GFM).

### Statistical analyses

Student’s t test was used to compare differences between two groups. *P* < 0.05 was considered statistically significant.

### Accession numbers

The transcriptome microarray data have been uploaded to ArrayExpress (ArrayExpress, RRID:SCR_002964) under accession number E-MTAB-8605.

## Supplementary information

Supplementary data

Suplementary data table S13.1

Suplementary data table S13.2

Suplementary data table S13.3

Uncropted WB

Reproducibility Checklist

## Data Availability

The transcriptome microarray data have been uploaded to ArrayExpress (ArrayExpress, RRID:SCR_002964) under accession number E-MTAB-8605.
