# Supplementary material for: A noncanonical response to replication stress protects genome stability through ROS production, in an adaptive manner
Source: Cell Death Differ. 2023 Mar 3;30(5):1349–65. doi: 10.1038/s41418-023-01141-0 (PMC10154342; doi:10.1038/s41418-023-01141-0)
Supplement: Supplementary file 1 — Supplementary data [file 41418_2023_1141_MOESM1_ESM.docx]

**Supplemental information.**

**A noncanonical response to replication stress protects genome stability through ROS production, in an adaptive manner**

Sandrine Ragu1,2, Nathalie Droin3, Gabriel Matos-Rodrigues1,2, Aurélia Barascu1#, Sylvain Caillat4,Gabriella Zarkovic5, Capucine Siberchicot6,Elodie Dardillac1,2, Camille Gelot1#, Josée guirouilh-Barbat1,2, J. Pablo Radicella6, Alexander A. Ishchenko5, Jean-Luc Ravanat4, Eric Solary3 and Bernard S. Lopez1,2*

**S1. Individual experiments showing HU-induced ROS.**

**S2. Impact of NAC and of the level of ambient oxygen.**

**S3. Impact of prolonged exposure to HU on ROS production in primary human fibroblasts.**

**S4. Impact of the HU or APH on cell cycle distribution and BrdU pulse incorporation.**

**S5. Effect of HU on plasmid transfection efficiency.**

**S6. Effect of HU exposure on OGG1, MTH1 and APE1.**

**S7. Expression of detoxification proteins upon HU or APH exposure.**

**S8. NRF2-controlled genes upon HU exposure.**

**S9. Efficiency of DUOX1 and DUOX2 mRNA silencing.**

**S10. Impact of ATM and p53 inhibitors on RIR production.**

**S11. Activation of p53 and γ-H2AX based on different doses of HU.**

**S12. Activation of p53 and γ-H2AX based on different doses of APH or CPT.**

**S13. Microarray analysis comparing primary human fibroblasts treated with HU and untreated primary fibroblasts.**

**S14. Impact of PARP inhibitors on RIR production.**

**S15. Table S12: List of primers used for SYBR real-time RT–PCR.**

**Table S13A1: Microarray scores (Excel file)**

**Table S13A2: DAVID_Downregulated genes (Excel file)**

**Table S13A3: DAVID_Upregulated genes (Excel file)**

**Supplementary data S1**

**Figure S1. Individual experiments showing HU-induced ROS.**

**Supplementary data S2**

**Figure S2. A. F.** Effect of NAC on RIR levels in primary fibroblasts. In all of the above experiments, data from three independent experiments are presented as the mean (± SEM) level of ROS production normalized to that of the control. **B.** Impact of NAC and 250 µM HU on the cell cycle and DNA synthesis.Upper panel: one example of FACS analysis. Lower panel: quantification from 4 independent experiments. **B.** Impact of the level of ambient oxygen.ROS induced by low doses of HU or APH are independent of ambient oxygen. Cells maintained at 20% O2 or 3% O2 were treated with increasing concentrations of HU (left) or APH (right) for 72 h, and DCF fluorescence was measured.

**Supplementary data S3.**

**Impact of prolonged exposure to HU on ROS production in primary human fibroblasts**

**Figure S3.** Impact of prolonged exposure to HU on ROS production in primary human fibroblasts. Primary fibroblasts were treated with HU (250 μM) for 2, 3, 4 or 7 days, and DCF intensity was measured.

**Supplementary data S4.**

**Impact of the HU or APH on cell cycle distribution and BrdU pulse incorporation.**

**A.**

**B.**

**C.**

**Figure S4. A.** Impact of the HU dose on cell cycle distribution and BrdU pulse incorporation. **B.** Impact of 0.6 µM APH on cell cycle distribution and BrdU pulse incorporation. **C.** Impact of ATM and p53 inhibitors on the cell cycle upon HU exposure.

**Supplementary data S5**

**Effect of HU on plasmid transfection efficiency.**

**Figure S5: Impact of HU on plasmid transfection efficiency.** A plasmid encoding the GFP gene was transfected (3 µg) into U2OS (left panels) or SV40-transformed (RG37) fibroblasts that were exposed (lower panels) (or not; middle panels) to 250 µM HU for 3 days before transfection. The percentage of fluorescent cells (P2 in red in the panels) was monitored by FACS analysis to determine transfection efficiency.

**Supplementary data S6**


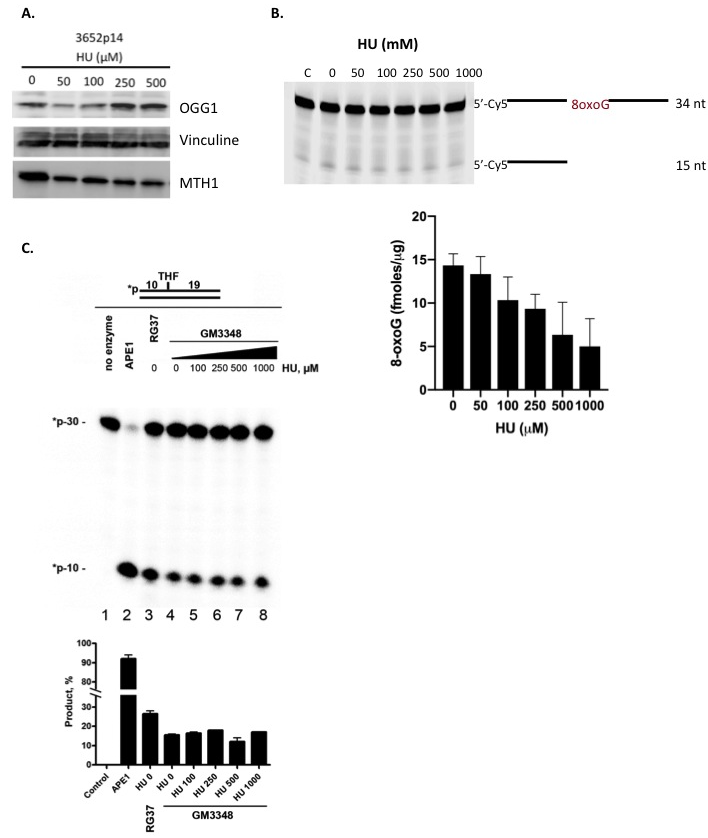


GM3652

**Figure S6. Impact of HU exposure on OGG1, MTH1 and APE1. A. Western blot** of OGG1 and MTH1. **B. 8-OxoG DNA glycosylase activity in whole-cell extracts from cultures treated with HU.** 8-OxoG excision was determined in whole-cell extracts by the cleavage assay on an oligonucleotide harboring an 8-oxoG residue (upper panel). Values correspond to the mean and SEM of three assays (lower panel). **C. APE1 activity.** AP endonuclease activities in RG37 (SV40-transformed human fibroblasts) and GM3348 (human primary fibroblasts) cell-free extracts after HU treatments. (Upper panel) PAGE analysis of AP endonuclease activities on a double-stranded THF•C oligonucleotide substrate containing a single tetrahydrofuran (THF, a stable abasic site analog). *p-10 band on the polyacrylamide gel corresponds to the expected AP endonuclease activity. (Lower panel) Comparison of AP endonuclease activities in the experiments presented in the upper panel. The error bars represent the standard deviation (n=3). For details, see the *Supplementary Materials and Methods*.

***Supplementary Materials and Methods S2***

**8-OxoG DNA glycosylase assay**. Cell pellets were sonicated in 20 mM Tris-HCl (pH 7.5), 250 mM NaCl, and 1 mM EDTA containing a cocktail of apoprotinin, antipain, and leupeptin (0.8 µg/µl each). The homogenate was centrifuged at 20,000 g for 30 min at 4°C, and aliquots of the supernatant were stored at -80°C. Protein content was measured using a Bio-Rad Assay Kit (Bio-Rad Laboratories, Richmond, CA) with BSA as a standard. A 34-mer oligonucleotide containing an 8-oxoG at position 16 and labeled at the 5’ end with Cy5 was hybridized to its complementary oligonucleotide containing a cytosine opposite the lesion. In a standard reaction mixture, protein extracts (20 or 10 µg in a final volume of 10 µl) were added to a 20 µl reaction mixture containing 150 fmoles of the 8-oxoG:C labeled duplex in 20 mM Tris-HCl (pH 7.1), 1 mM EDTA, 200 mM NaCl, 1 mg/ml BSA and 5% glycerol. After incubation for 1 h at 37°C, NaOH (0.1 N final concentration) was added, and the mixture was further incubated for 15 min at 37°C and stopped by adding 4 µl of formamide dye and heating for 5 min at 95°C. The products were resolved by denaturing (7 M urea)-20% polyacrylamide gel electrophoresis. The gels were scanned, and band intensities were quantified using a Typhoon Phosphorimager (GE Healthcare).

**APE1 Activity assay.**

***Preparation of cell extracts.*** Briefly, the cell pellet was washed in ice-cold PBS and incubated for 15 min at 4°C with shaking in 3 volumes of lysis buffer containing 0.5 M KCl, 80 mM HEPES (pH 7.6), 0.1 mM EDTA, 2 mM DTT, 0.3% NP-40 and protease inhibitor cocktail (Complete EDTA-free, Roche). After centrifugation at 20000 rpm for 1 h at 4°C, the supernatants were collected and stored in 50% glycerol at -20°C for immediate use or at -80°C for longer storage. Protein concentration was determined by the Bradford assay.

***Oligodeoxyribonucleotide duplexes.*** Oligodeoxyribonucleotides were purchased from Eurogentec (Seraing, Belgium) and included the following: d(TGACTGCATAXGCATGTAGACGATGTGCAT) 30-mer, where X is either thymidine or tetrahydrofuranyl (THF), and complementary oligonucleotides. Oligonucleotides containing THF were 5’-end labeled by T4 polynucleotide kinase (New England Biolabs, OZYME France) in the presence of [γ-32P]-ATP (3,000 Ci•mmol-1) (PerkinElmer SAS, France) as recommended by the manufacturer. Complimentary oligonucleotides were annealed by heating at 65°C for 5 min and cooling slowly to room temperature for 1 h. The resulting duplexes are referred to as THF•C and T•A, containing either dC opposite the THF or dA opposite the T, respectively.

***AP endonuclease assays.*** The standard assay mixture for AP endonuclease activity (20 µl final volume) contained 0.2 pmol of the 5´-[32P]-end labeled THF•C and 2 pmol of cold T•A oligonucleotide duplexes in 20 mM Tris/HCl (pH 7.6), 50 mM KCl, 0.5 mM MgCl2, 100 µg bovine serum albumin/ml, and either 12.5 µg/ml cell-free extract or 2.5 nM recombinant human APE1 enzyme (laboratory stock). Incubations were carried out at 37°C for 10 min. Desalted reaction products were heated at 65°C for 3 min and separated by electrophoresis in denaturing 20% (w/v) polyacrylamide gels (20:1, 7 M urea, 0.5x TBE). The gels were exposed to a Fuji FLA-3000 Phosphor Screen and analyzed using ImageGauge V3.12 software.

**Supplementary data S7.**

**Expression of detoxification proteins upon HU or APH exposure.**

**Figure S7.** Induction of detoxication proteins by HU or APH.

**Supplementary data S8.**

**NRF2-controlled genes upon HU exposure.**


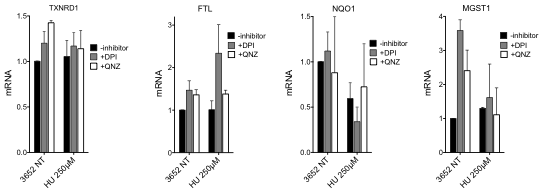


**Figure S8.** NRF2-controlled genes were not induced by HU treatment in primary fibroblasts.

**Supplemental material S9.**

**Efficiency of silencing DUOX1 and DUOX2 mRNA silencing.**

**A.**

**B.**

**Figure S9**. Efficiency of silencing DUOX1 and DUOX2 mRNAs. siRNA against DUOX1 or DUOX2 or a control (scrambled) siRNA was transfected into primary fibroblasts, and HU was added (or not). After 72 h, the cells were harvested, and DUOX1 and DUOX2 mRNA expression was analyzed by real-time qRT-PCR. The data represent the relative expression of DUOX mRNAs normalized to that of the control (scrambled siRNA) after all samples were normalized to beta-actin mRNA expression. Two different siRNAs were assayed. **A.** siDUOX1(1) and siDUOX1(2). **B.** siDUOX1(2) and siDUOX2(2).

**Supplemental material S10.**

**Impact of ATM and p53 inhibitors on RIR production**

**
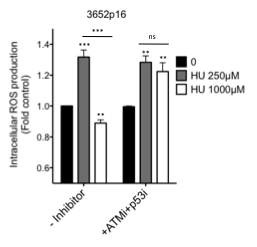
**

**Figure S10. Impact of ATM and p53 inhibitors on RIR production.** Effect of Ku55933 (an ATM inhibitor) and pifithrin (a p53 inhibitor) on RIR production induced by hydroxyurea (low and high concentrations) in primary fibroblast strains.

**Supplemental material S11**

**Activation of p53 and γ-H2AX based on different doses of HU.**

250


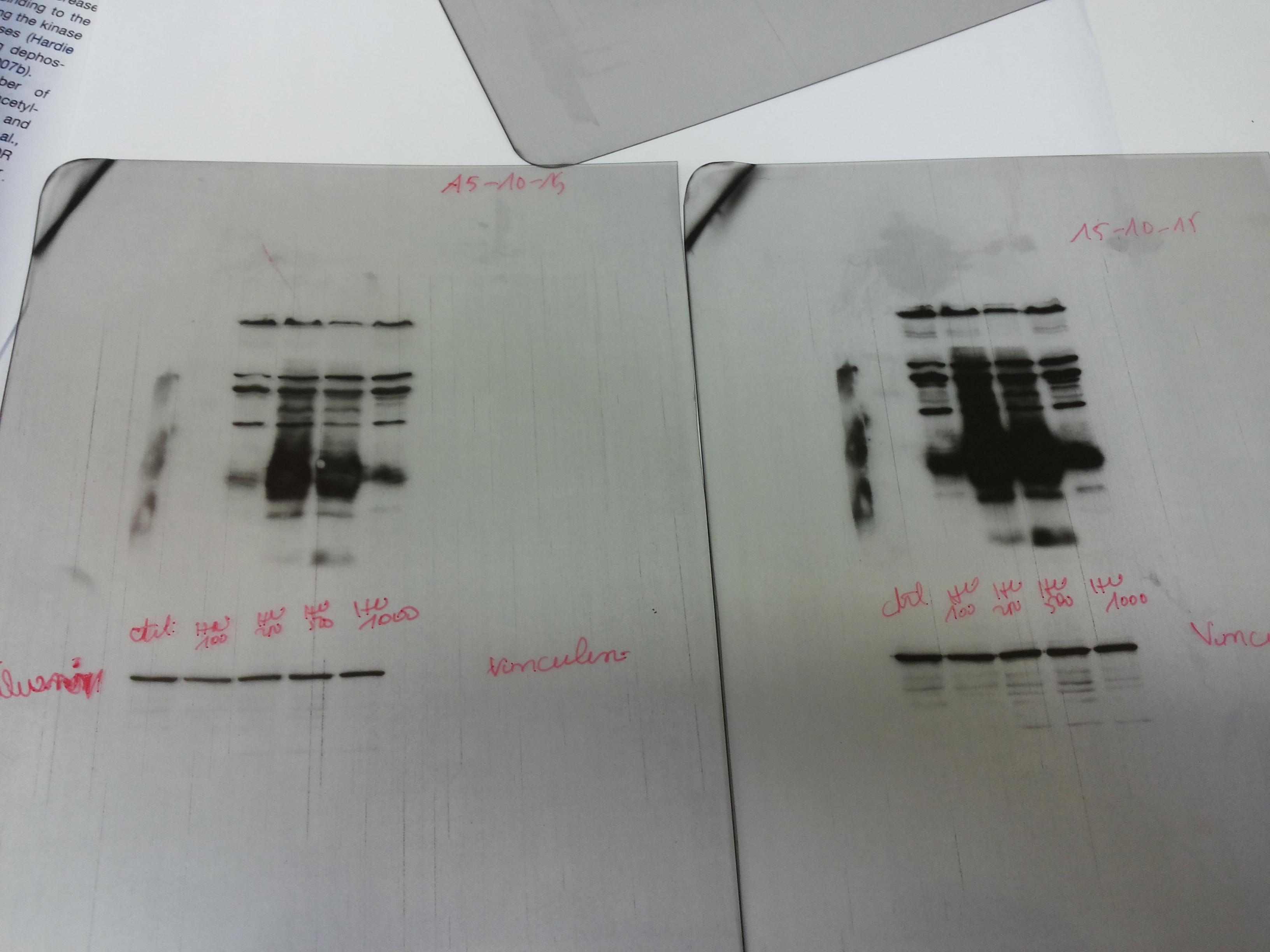

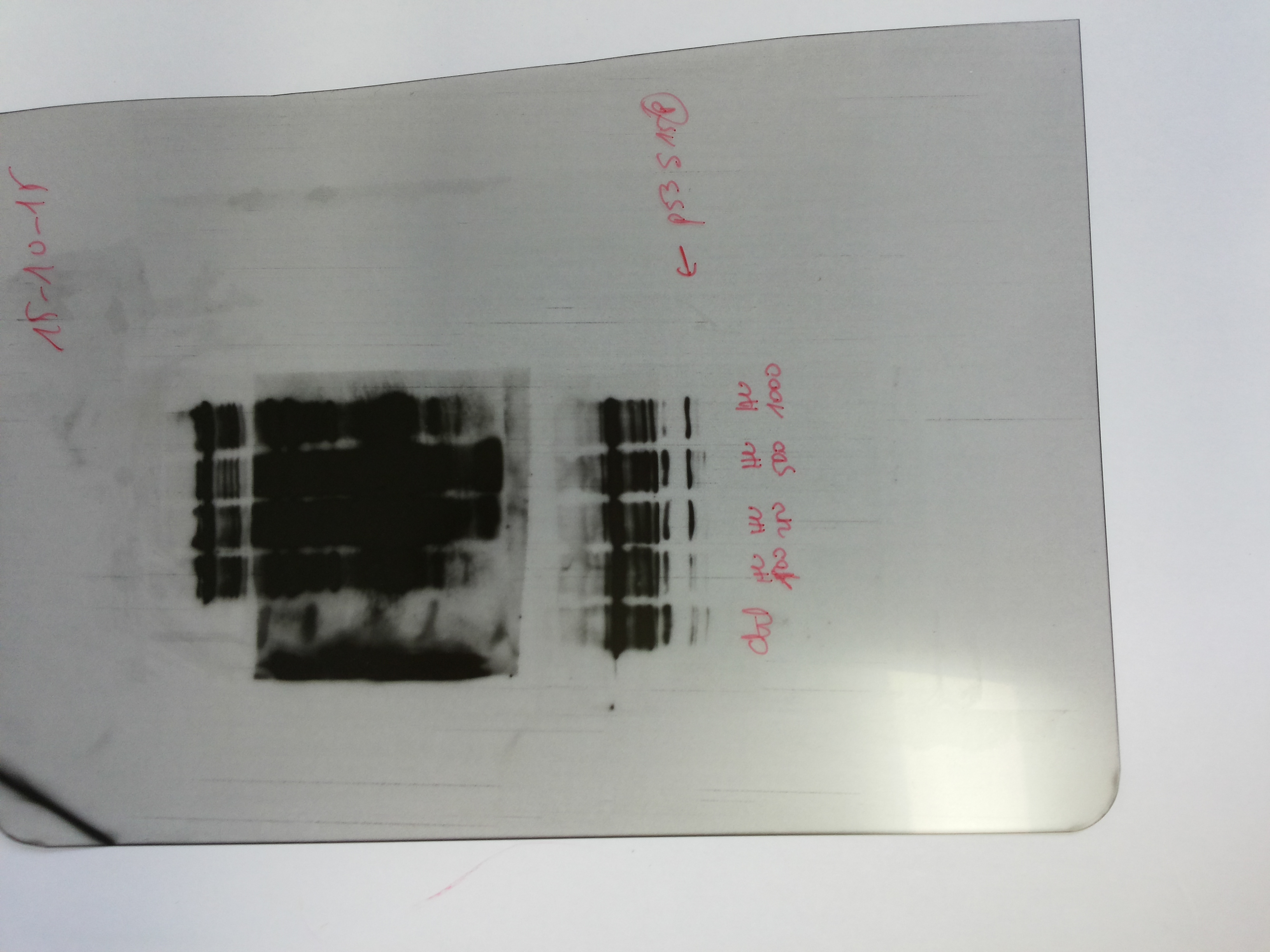


HU(µM):

100

0

500

1000

Vinculine

p-S15-p53


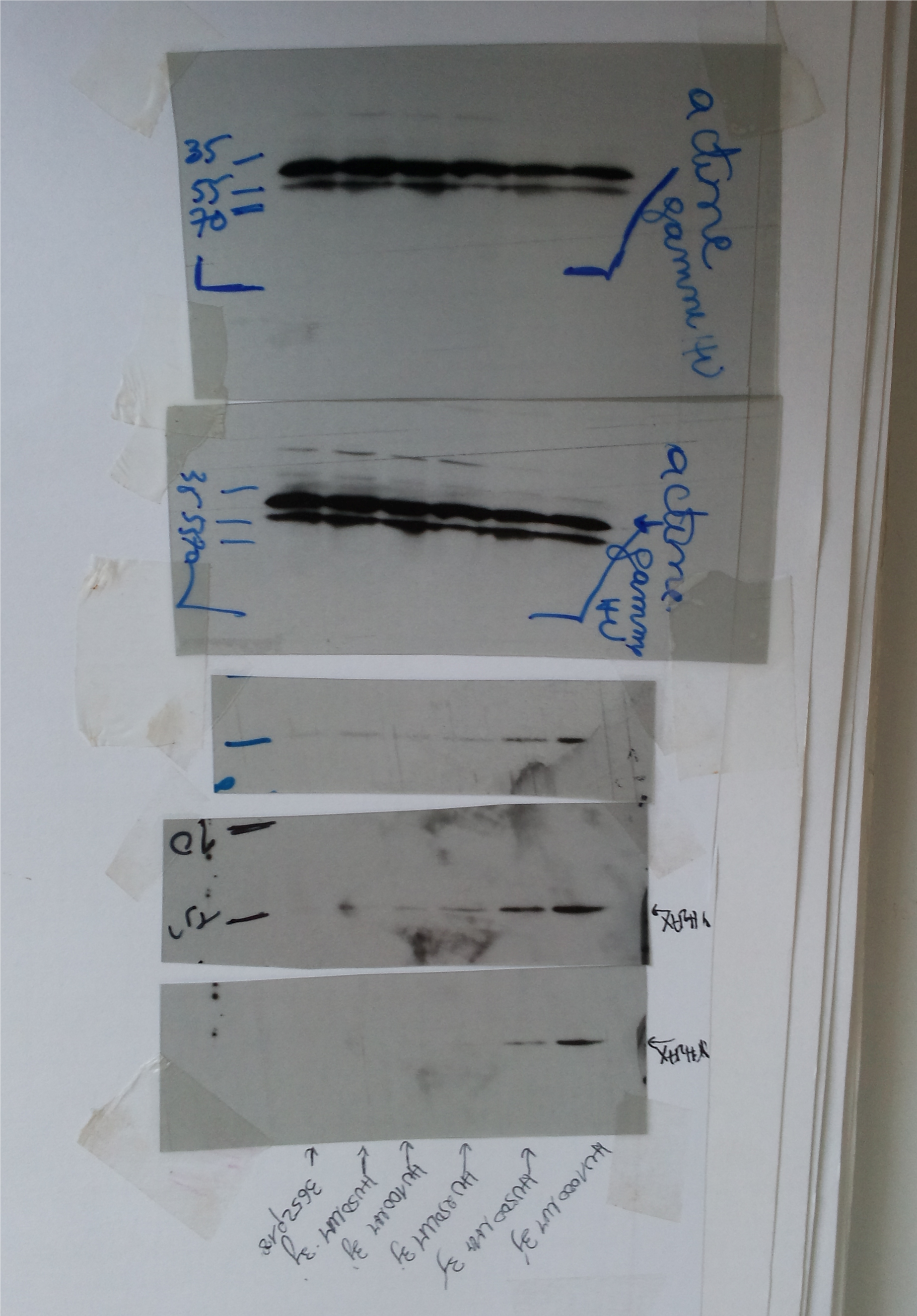

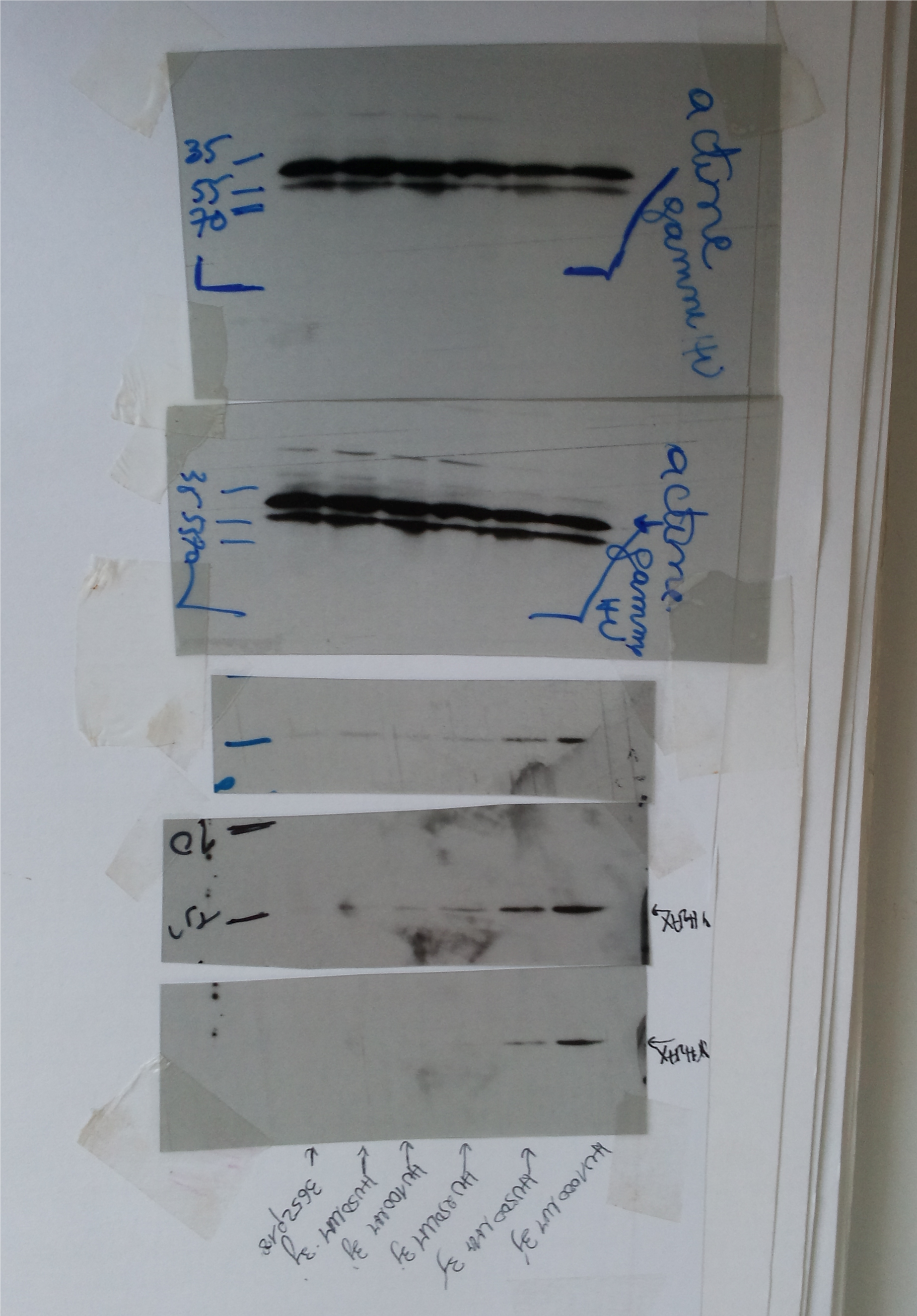


γ−H2AX

Actine

GM03652

Primary Fibroblasts

**Figure S11.** Immunoblots of phosphorylated p53 and γH2AX levels in untreated and HU-treated primary fibroblasts after 72 h of treatment.

**Supplemental material S12.**

**Figure S12.** Immunoblots of phosphorylated p53 and γH2AX levels in APH (**A**) and CPT (**B**) treated primary fibroblasts after 72 h of treatment. Two different exposures are shown for p-S15-p53.

**Supplemental material S13.**

**Microarray analysis**

**Supplementary data S13A.**

We performed a microarray analysis comparing primary human fibroblasts treated with 250 μM HU for three days with untreated cells. We used a cutoff of log2 (fold change - FC) > 0.5 and < -0.5 and an adjusted p-value of 0.05, and 152 down- and 416 upregulated targets were found after HU treatment.

**Table S13A1: Microarray scores (see Excel table S9A.1)**

**Table S13A2: DAVID_Downregulated genes (see Excel table S9A.2)**

**Table S13A3: DAVID_Upregulated genes (see Excel table S9A.3)**

**Supplementary data S13B: Upregulation of genes controlled by p65 (RelA) after HU.**

We searched for transcription factors that could regulate the expression of genes included in our microarray. The analysis revealed p65, a member of the NF-kB signaling pathway, as a possible transcription factor activated after 250 μM HU treatment.

List of 69 NF-kB/p65-controlled genes upregulated after HU

NOG, SNORA12, IL4I1, GRIN3B, CPEB1, GDNF, GSTM5, IL11, LNX1, ARHGAP22, TRIM47, SLC24A3, SPINT2, IL1B, NOS3, NRG1, C17ORF96, KDELR3, H1F0, ICAM1, CAMK1G, EFNB1, NUDT14, RELB, TNFRSF14, SNORD96B, SNORD96A, HMGA2, PKIA, UCN2, TLCD1, PTGDS, KRT17, BTG2, CD82, CTSH, CCL2, PFKFB4, NFKBIE, NFKBIA, ATP6V1G2, NFIX, GPR68, SFN, DENND2D, FAM46C, LAPTM5, TEK, NUMB, HAAO, OBSL1, NEDD4L, NDRG2, DCLK1, C17ORF82, MAFF, IL6, NFE2, EEF1A2, PODXL, BIRC3, COG4, LAMA4, GMFG, SVIL, NOTCH4, FAM43B, MAP6, CEND1

**Supplementary data S13C: Upregulation of the NF-κB pathway in CD3-positive T cells (proliferative) in peripheral blood samples from four CMML patients chronically treated with HU**


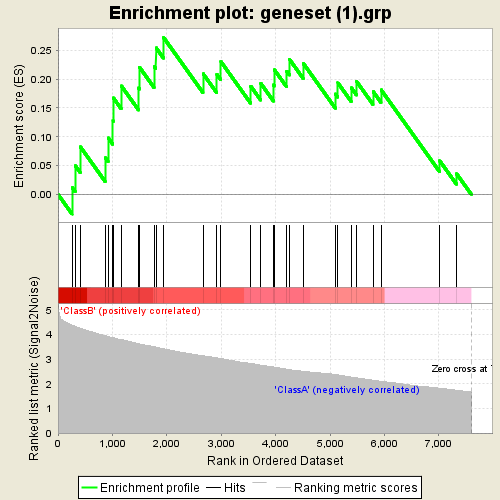


Patient#1

FDR: 0.1431694


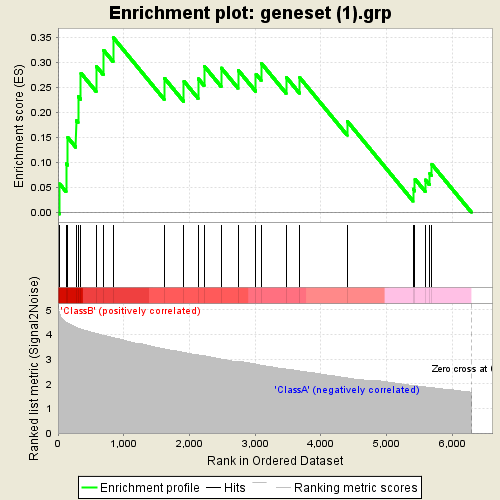


Patient#2

FDR: 0.033557046


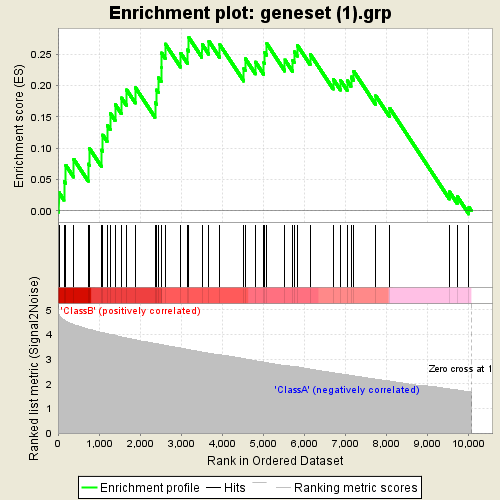


Patient#3

FDR: 0.055265903


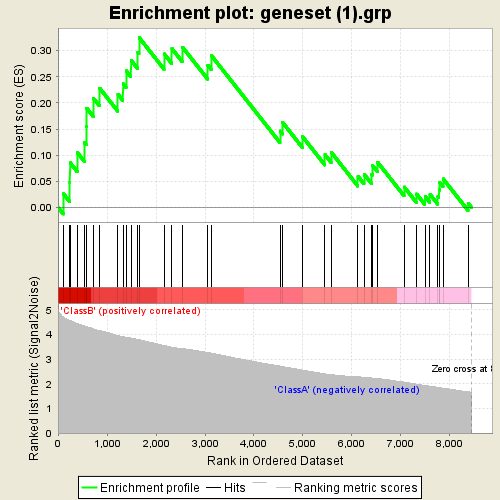


Patient#4

FDR: 0.01268499

**Figure S13C.** Enrichment of NF-B-controlled genes (green curves, GSEA) from its upregulation revealed by RNA-seq analysis of CD3-positive T cells from 4 different CMML patients comparing samples before and after chronic hydroxyurea treatment.

**Supplemental material S14.**

**Impact of PARP inhibitors on RIR production**


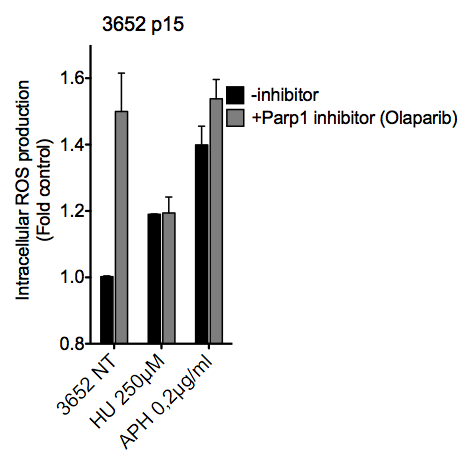

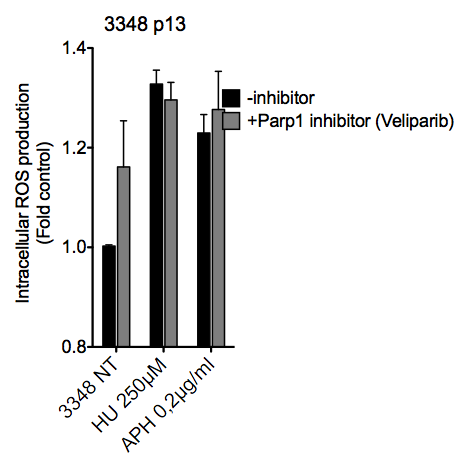


**Figure S14**. Impact of PARP inhibitors on RIR production. Effect of olaparib and veliparib (a PARP1 inhibitor) on RIR production induced by hydroxyurea and aphidicolin in primary fibroblast strains.

**Supplementary data S15.**

**Table S15**: List of primers used for SYBR real-time RT–PCR.

| **Gene Symbol** | **Forward primer 5'-3'** | **Reverse primer 5'-3'** |
| --- | --- | --- |
| SMC4 | GGCTGTATGGGCGAAAAAGAT | TTGTGGCTTGATCCAAGTTGT |
| LMB1 | GAAAAAGACAACTCTCGTCGCA | GTAAGCACTGATTTCCATGTCCA |
| MCM3 | TCAGAGAGATTACCTGGACTTCC | TCAGCCGGTATTGGTTGTCAC |
| HIST1H3F | TACTGTCGCCCTCCGTGAAA | CACCAGGTAAGCCTCGCAG |
| TOP2B | TTGGACAGCTTTTAACATCCAGT | GCACCATAACCATTACGACCAC |
| CCL2 | CAGCCAGATGCAATCAATGCC | TGGAATCCTGAACCCACTTCT |
| CXCL14 | CGCTACAGCGACGTGAAGAA | GTTCCAGGCGTTGTACCAC |
| CDKN1A (p21) | TGTCCGTCAGAACCCATGC | AAAGTCGAAGTTCCATCGCTC |
| IL4I1 | TGATGTCCGAGGATGGCTTCT | TGTACTGGAGTCTGTCGCTGA |
| CD82 | TGTCCTGCAAACCTCCTCCA | CCATGAGCATAGTGACTGCCC |
| SOD2 | GCTCCGGTTTTGGGGTATCTG | GCGTTGATGTGAGGTTCCAG |
| IL6 | CCTGAACCTTCCAAAGATGGC | TTCACCAGGCAAGTCTCCTCA |
| GAPDH | ACAACTTTGGTATCGTGGAAGG | GCCATCACGCCACAGTTTC |
| ACTB | TGACCCAGATCATGTTTGAGA | TACGGCCAGAGGCGTACAGG |
