# Supplementary figures and images for: A noncanonical response to replication stress protects genome stability through ROS production, in an adaptive manner
Source: Cell Death Differ. 2023 Mar 3;30(5):1349–65. doi: 10.1038/s41418-023-01141-0 (PMC10154342; doi:10.1038/s41418-023-01141-0)

Fig. 4B

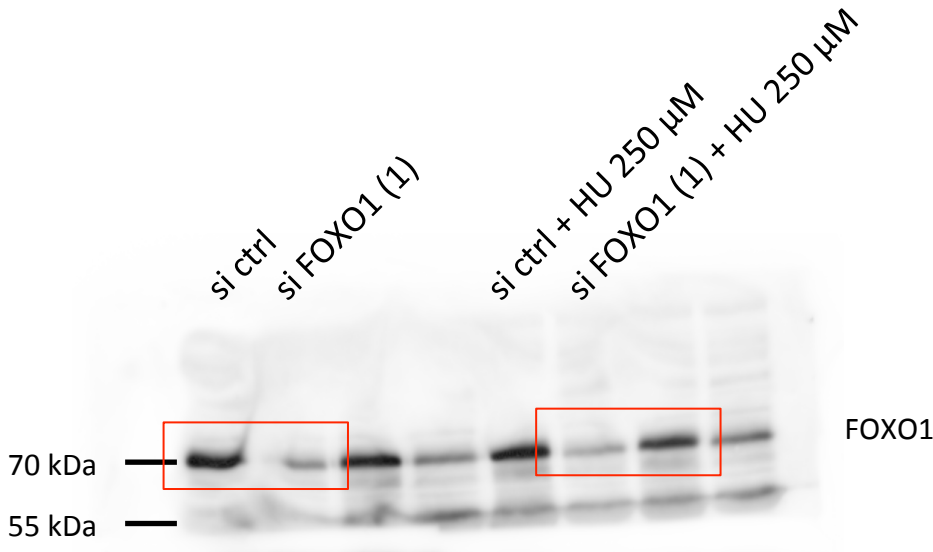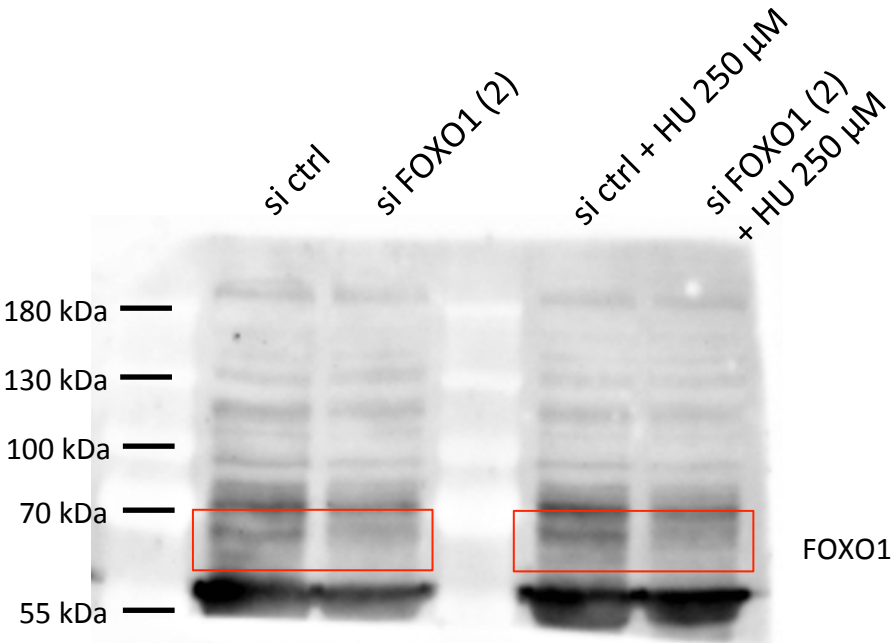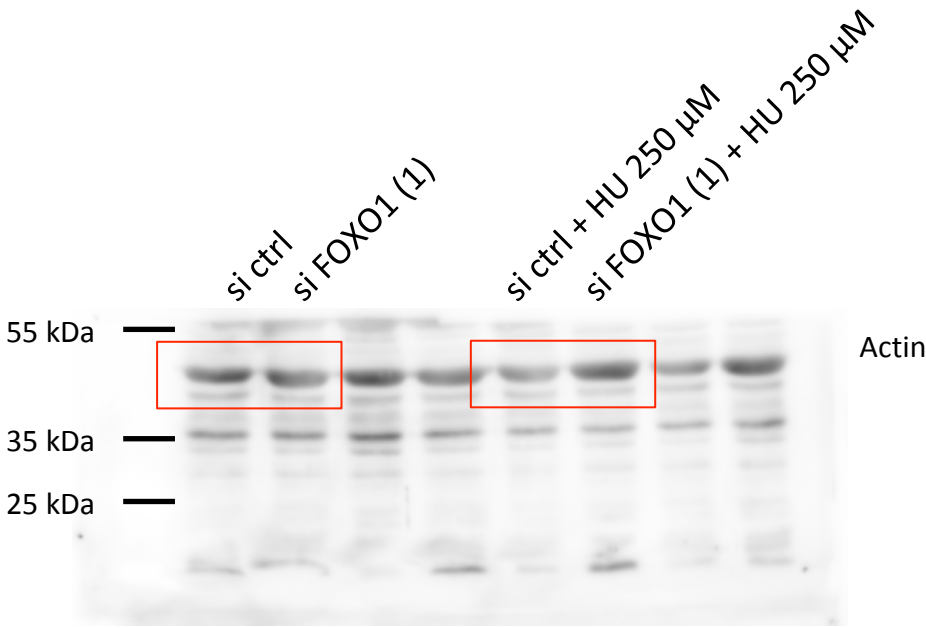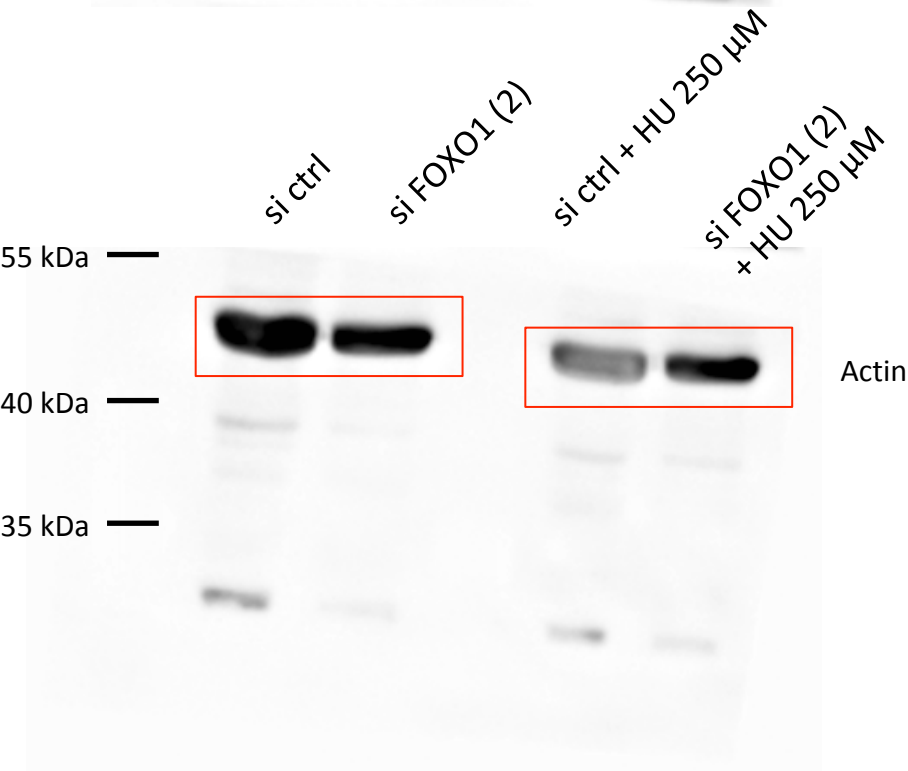

Figure 5

Fig. 5E

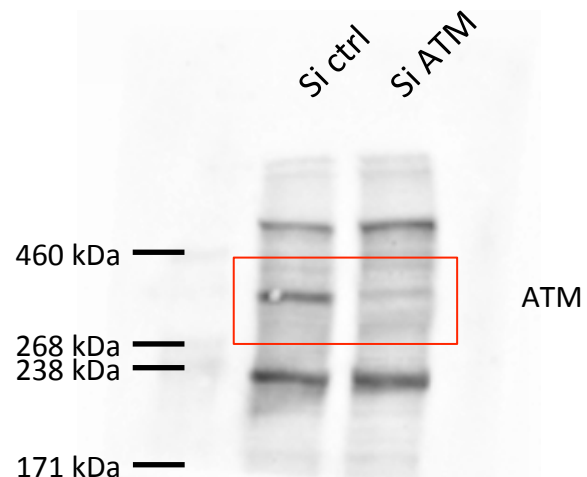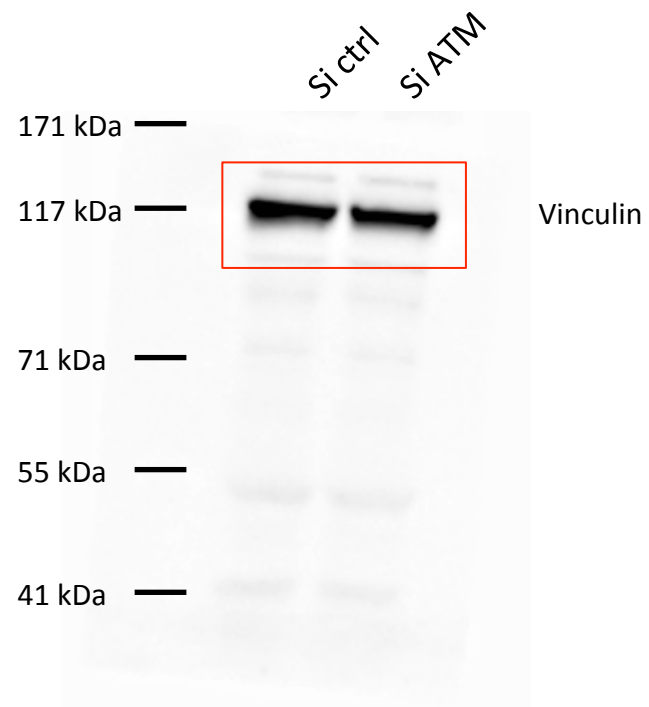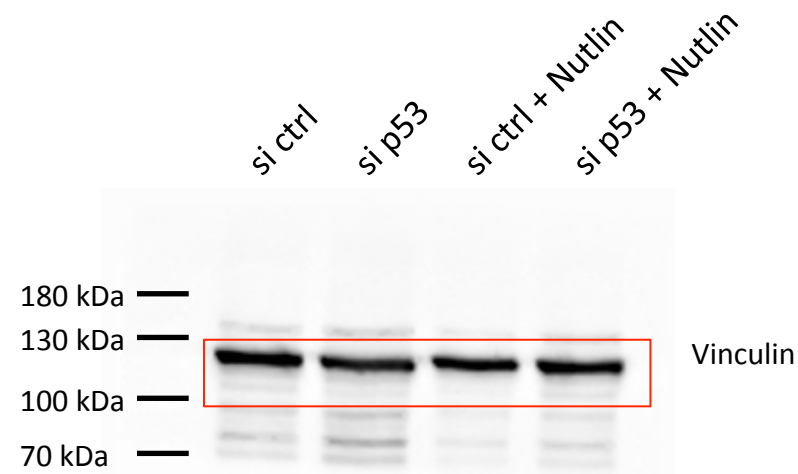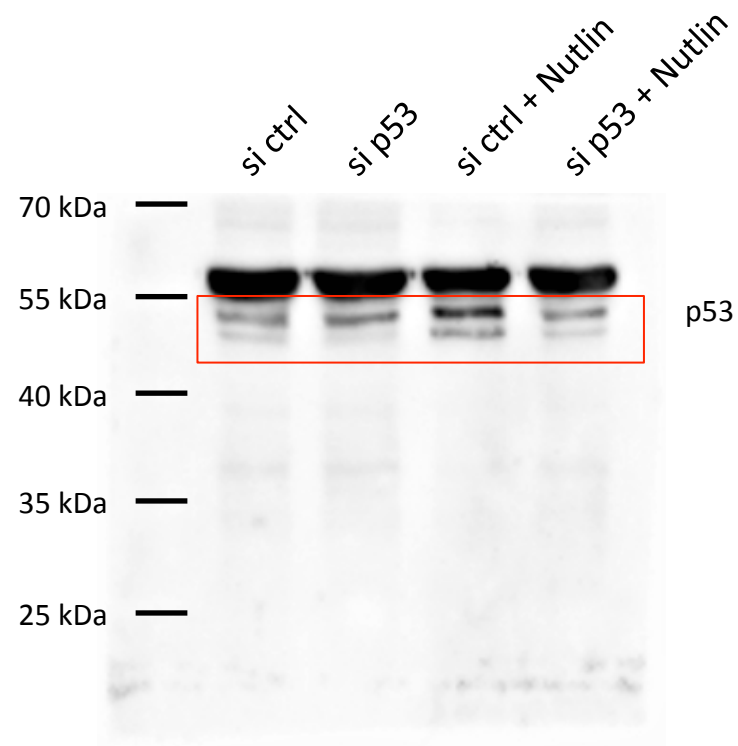

Figure 7

Fig. 7A

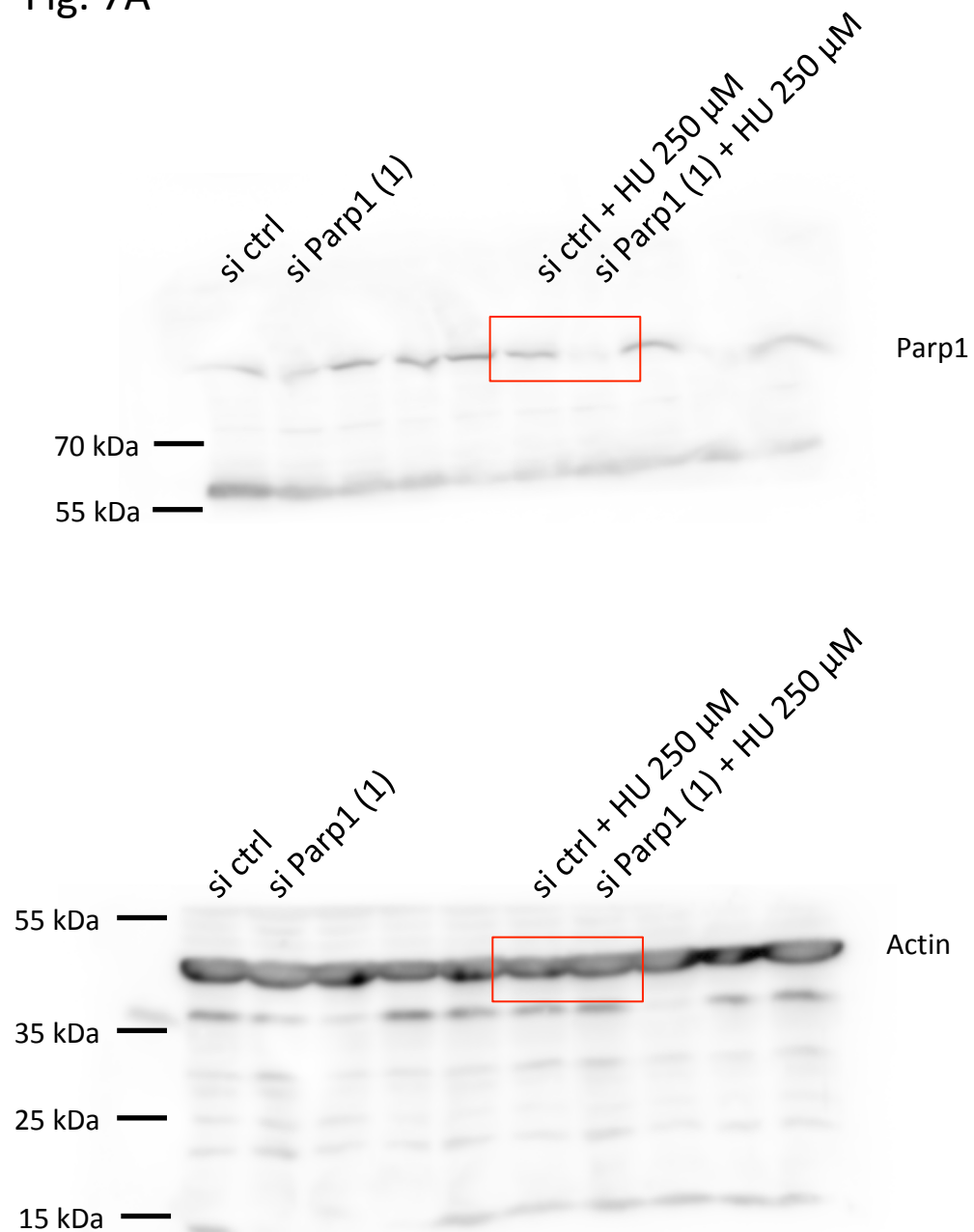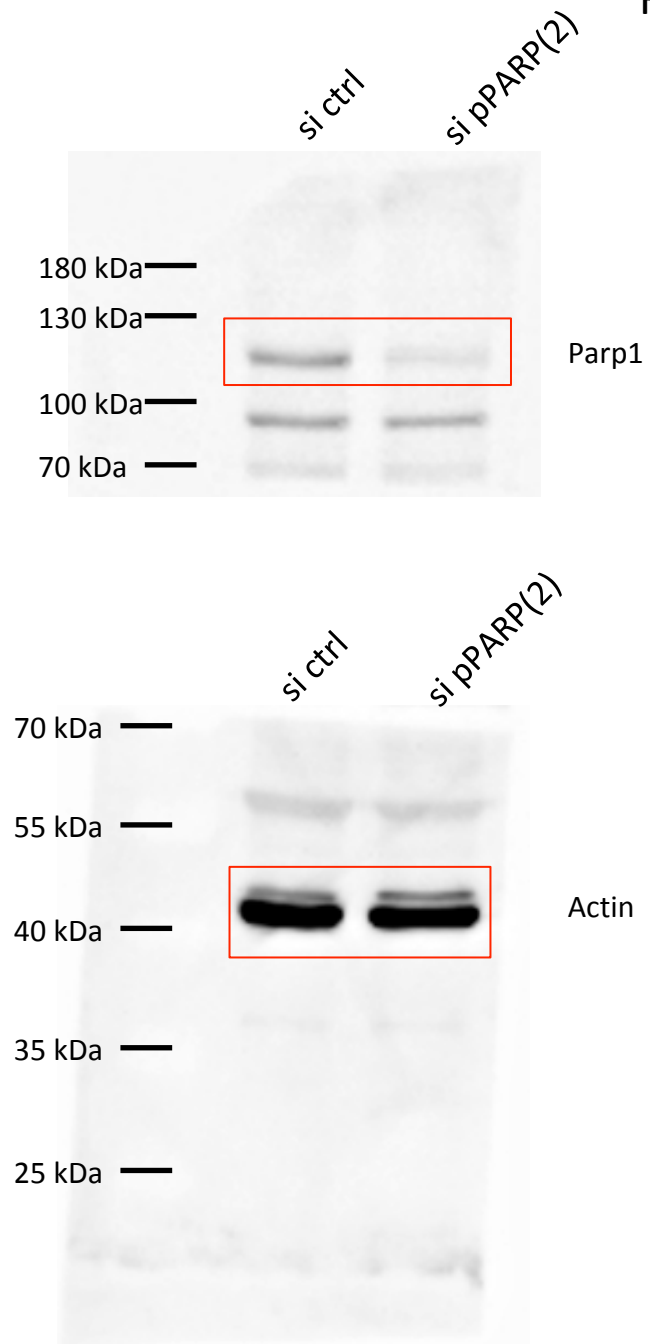

Fig. S7

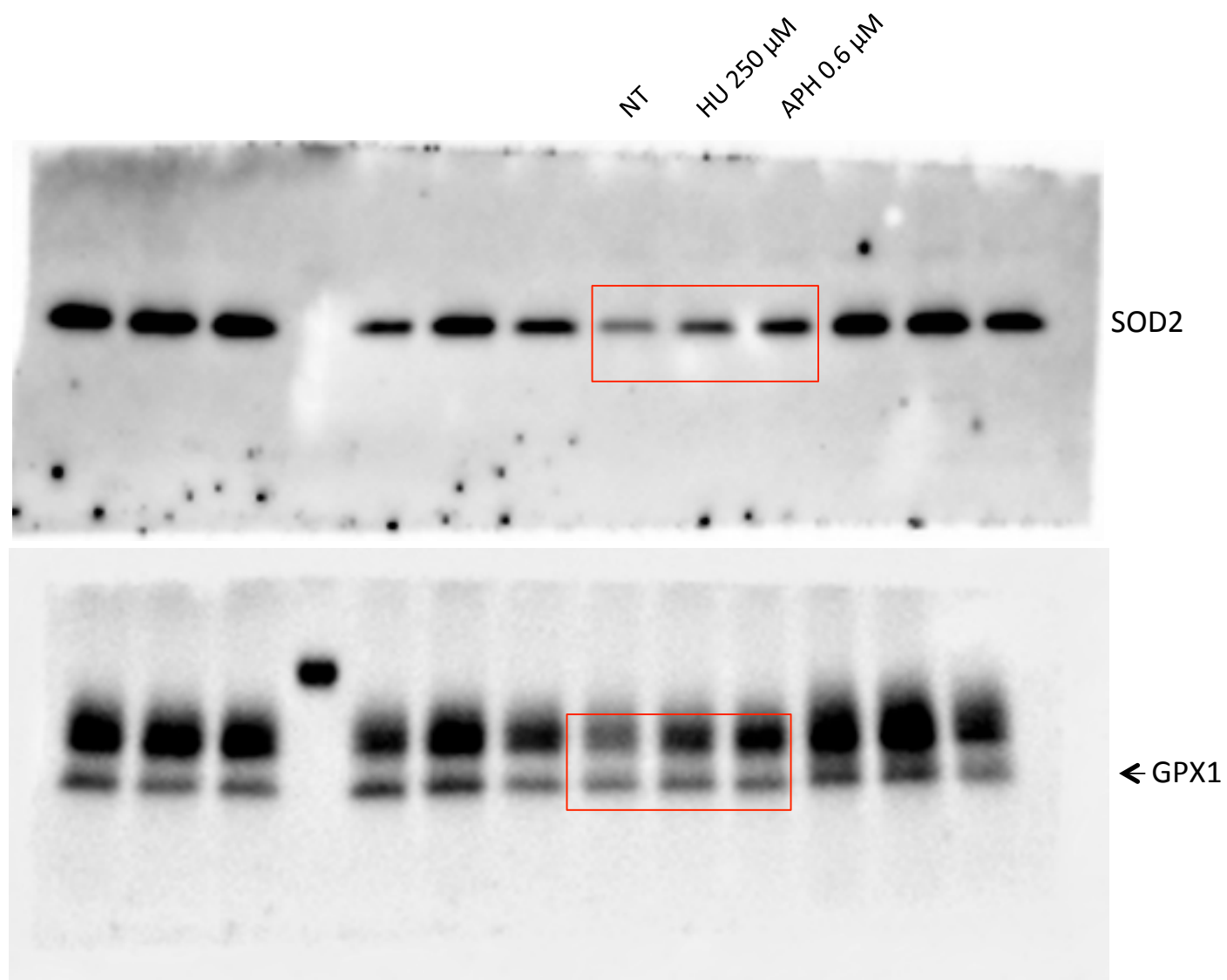

Fig. S7 (suite)

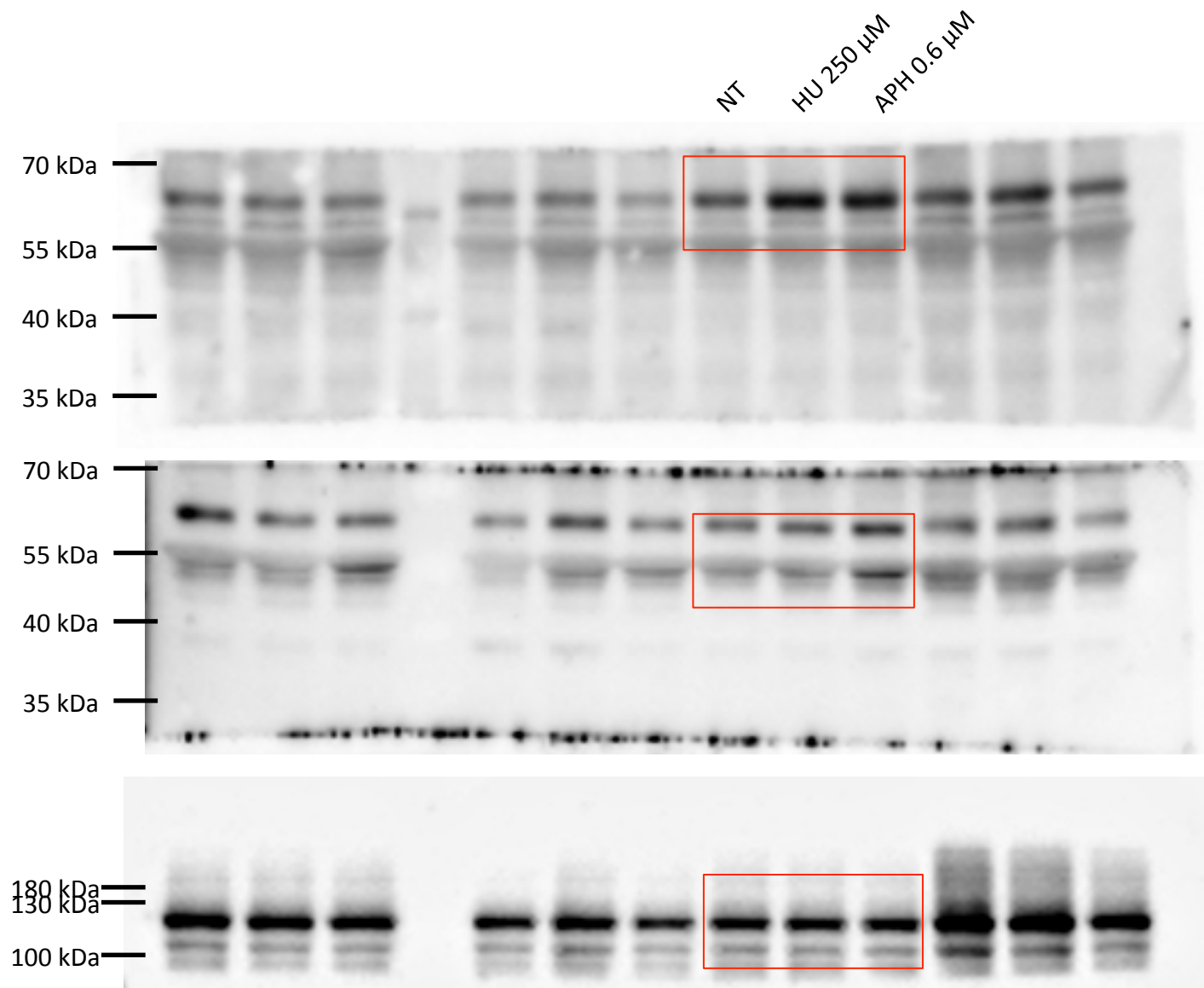

Fig. S12

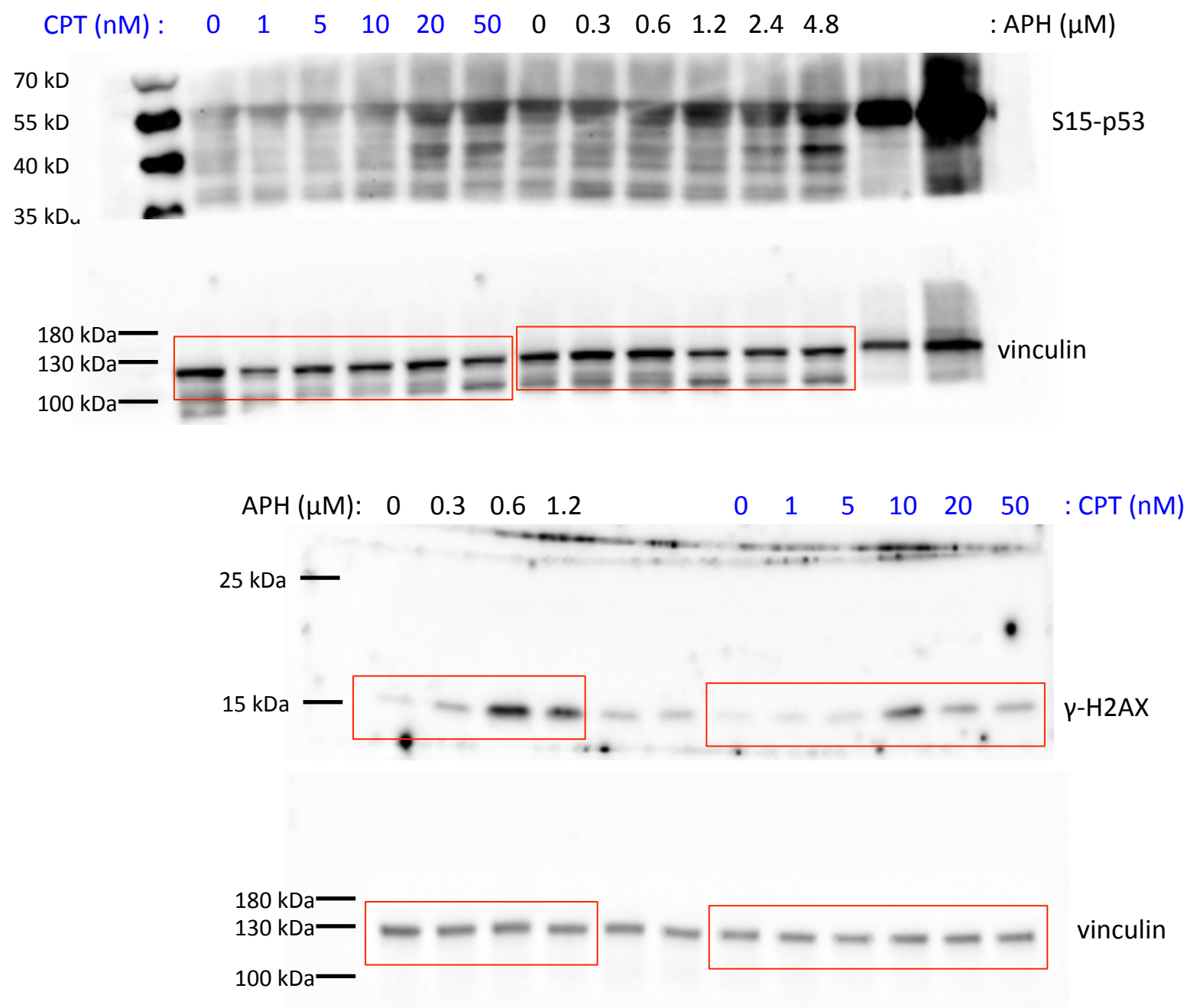

Supplement: Supplementary file 5 — Uncropted WB [file 41418_2023_1141_MOESM5_ESM.pdf]
